# Exploratory meta-analysis of the effect of music intervention on arousal promotion in patients with disorders of consciousness: evidence from controlled studies

**DOI:** 10.3389/fnins.2026.1831090

**Published:** 2026-05-08

**Authors:** Jiayi Gu, Chengjuan Li, Wei Long, Siqin Zeng, Xiaoying Zhang

**Affiliations:** 1 Department of Rehabilitation, Hunan Provincial People's Hospital, The First Affiliated Hospital of Hunan Normal University, Changsha, China; 2 Department of Rehabilitation, The First Hospital of Changsha, The Affiliated Changsha Hospital of Xiangya School of Medicine, Central South University, Changsha, China; 3 Department of Neurological Rehabilitation, Hunan Rehabilitation Hospital, Changsha, China; 4 School of Rehabilitation Medicine, Capital Medical University, Beijing, China; 5 Music Therapy Center, China Rehabilitation Research Center, Beijing, China

**Keywords:** arousal, disorders of consciousness, meta-analysis, music intervention, rehabilitation

## Abstract

**Introduction:**

Disorders of consciousness (DoC) are common disabling conditions following severe brain injury that impose a substantial burden on families and society. Music intervention, as a non-invasive stimulation method, has garnered increasing attention in the field of neurorehabilitation. However, evidence regarding its awakening effects in patients with DoC remains inconsistent. This study systematically reviewed controlled trials investigating the effects of music intervention on the level of consciousness in patients with DoC and performed an exploratory meta-analysis.

**Methods:**

We systematically searched five databases (PubMed, Cochrane Library, Web of Science, Embase, and CNKI) to identify controlled trials comparing the effects of music intervention and control conditions on the level of consciousness in patients with DoC. The Cochrane RoB 2.0 and ROBINS-I tools were used to assess risk of bias. Statistical analysis was performed using RevMan V5.4.1. The primary outcome was the standardized mean difference (SMD) in consciousness levels, calculated as the change in consciousness scores from baseline to endpoint between the music intervention and control groups.

**Results:**

Seven studies (four randomized controlled trials and three non-randomized studies) comprising 296 patients were included. The meta-analysis showed that the music intervention group had significantly greater improvement in consciousness level scores compared with the control group (SMD = 1.51, 95% CI: 0.57–2.44, *P* < 0.01), with high heterogeneity across studies (*I*^2^ = 91%). Sensitivity analysis indicated that heterogeneity decreased to a moderate level (*I*^2^ = 72%) after excluding one study involving interactive music therapy and decreased further (*I*^2^ = 34%) after excluding studies with short intervention durations. The GRADE certainty of the evidence was rated very low.

**Conclusion:**

Music intervention may improve the level of consciousness in patients with DoC. However, the existing evidence is highly heterogeneous. Intervention type (interactive vs. passive) and intervention intensity may represent potential sources of heterogeneity. Further high-quality studies are required to confirm these hypotheses.

## 1 Introduction

Disorders of consciousness (DoC) are pathological conditions characterized by impaired awareness resulting from severe brain injury, including coma, vegetative state (VS)/unresponsive wakefulness syndrome (UWS), and minimally conscious state (MCS; Giacino et al., 2018). With advances in acute and critical care medicine, the survival rate of patients with severe brain injury has significantly improved. However, a considerable proportion of these patients experience prolonged DoC. Epidemiological surveys indicate that the prevalence of DoC is approximately 0.2–6.1 per 100,000 people, which imposes substantial economic and caregiving burdens on families and society (van Erp et al., 2014). Therefore, exploring effective, safe, and economical interventions to promote recovery of consciousness in patients with DoC has become a critical clinical issue requiring urgent attention in the field of neurorehabilitation.

Music intervention, a non-invasive, low-cost, and easily implementable stimulation method, has garnered increasing attention in neurorehabilitation in recent years. The theoretical basis is that music, as a complex acoustic signal, can activate multimodal neural networks involved in emotional, memory, and cognitive processing, including brain regions closely associated with levels of consciousness, such as the prefrontal cortex, cingulate gyrus, and auditory cortex (Okumura et al., 2014; Särkämö et al., 2016). Previous studies have suggested that music intervention may positively influence the level of consciousness in patients with DoC through mechanisms that modulate neurotransmitter release, improve cerebral blood perfusion, and promote neural plasticity (O'Kelly et al., 2013; Castro et al., 2015). A neurophysiological study by (O'Kelly et al. 2013) further demonstrated that individualized music stimulation can induce significant changes in electroencephalographic activity in patients with DoC, suggesting that music may exert regulatory effects through preserved neural pathways.

Recently, several researchers have attempted to systematically evaluate the effects of music interventions on patients with DoC. (Grimm and Kreutz 2018) conducted a systematic review of quantitative studies, providing preliminary evidence that music intervention may improve behavioral scores in patients with DoC. However, this study provided only qualitative descriptions and did not quantitatively synthesize effect sizes. Subsequently, the same team published a systematic review of qualitative studies in 2021, further elucidating patients' behavioral response patterns during music intervention and the important role of environmental factors in treatment (Grimm and Kreutz, 2021). (Li et al. 2020) performed a meta-analysis of 11 studies, showing that music intervention had positive effects on certain functional and physiological indicators. However, the included studies comprised both randomized controlled trials and observational studies without stratified analysis by study design, and the strength of the evidence requires further validation. Within the broader population of acquired brain injury, a Cochrane systematic review by (Magee et al. 2017) indicated that music intervention has potential value in improving speech, motor, and other functions in patients with brain injury. However, this review did not specifically focus on the core outcome of “consciousness recovery.”

Notably, substantial heterogeneity within the patient population, including variations in etiology, baseline consciousness levels, disease duration, and diversity in music intervention protocols, may contribute to the variability observed across study findings. A scoping review by (Lancioni et al. 2021) further indicated that music interventions can be categorized into multiple types, including recorded, interactive, and response-contingent music, with different intervention types potentially influencing patient outcomes through distinct mechanisms. Previous reviews, owing to the limited number of included studies or the absence of stratified analyses by intervention type, have been unable to thoroughly investigate these sources of heterogeneity. Therefore, we anticipated that substantial statistical heterogeneity might emerge when pooling effect sizes in this study, which necessitated exploring potential sources of heterogeneity through qualitative comparison of study characteristics alongside quantitative analysis.

In summary, there remains a lack of systematic reviews based on the most recent evidence that specifically focus on awakening effects in patients with DoC and provide a quantitative synthesis of key outcome indicators. Most existing reviews either provide only qualitative descriptions or focus separately on quantitative and qualitative evidence, lacking systematic integration of both evidence types. In addition, they often fail to distinguish the risk of bias between randomized controlled trials (RCTs) and non-randomized studies (NRS) for stratified analysis and inadequately address the relationship between specific music intervention parameters and therapeutic efficacy. Given the inherent heterogeneity in both the patient with DoC population and intervention protocols, we anticipated that substantial statistical heterogeneity might emerge in pooled effect estimates. Accordingly, this study aimed to evaluate the awakening effects of music interventions in patients with DoC through a systematic review and exploratory meta-analysis. We systematically searched domestic and international databases and included both RCTs and NRS. The risk of bias was assessed using the Cochrane RoB 2.0 and ROBINS-I tools. Key outcome indicators, including consciousness level scores and consciousness recovery rates, were quantitatively synthesized. Potential sources of heterogeneity, such as study design, patient characteristics, and intervention features, were explored through qualitative comparison of study characteristics alongside quantitative analysis. The findings of this study are expected to provide evidence-based medical support for music rehabilitation therapy in patients with DoC and offer a reference for future clinical research and practice.

## 2 Methods

### 2.1 Protocol and registration

This systematic review was conducted in accordance with the Preferred Reporting Items for Systematic Reviews and Meta-Analyses (PRISMA) guidelines (Page et al., 2021).

**Systematic review registration:** PROSPERO-URL: https://www.crd.york.ac.uk/prospero/display_record.php?ID=CRD420251161710.

### 2.2 Search strategy and selection criteria

We systematically searched five Chinese and English databases: PubMed, the Cochrane Library, Web of Science, Embase, and CNKI. A combination of Medical Subject Headings terms and free-text words was used. Search terms included “Disorders of Consciousness” OR “Persistent Vegetative State” AND “music therapy” OR “music” OR “music intervention” OR “music stimulation” OR “music exposure” OR “music-based intervention.” The search timeframe extended from database inception to October 2025. We also supplemented the search by reviewing reference lists of included studies and searching for gray literature. Attachment 1 presents the complete search strategy.

### 2.3 Inclusion criteria

P (Participants): Patients with a definitive diagnosis of DoC, including but not limited to coma, vegetative state/UWS, MCS, and chronic DoC.

Diagnostic Criteria: Diagnosis must be based on recognized clinical behavioral scales such as the Coma Recovery Scale, Glasgow Coma Scale, and UWS Score. Studies must explicitly report the diagnostic criteria used.

Demographic Characteristics: No restrictions were imposed on patient age, sex, or etiology (e.g., traumatic brain injury, stroke, or hypoxic encephalopathy). However, these factors were considered potential sources of heterogeneity during the analysis.

I (Interventions): The experimental group received any form of music-based intervention, including receptive music therapy, recreational music therapy, improvisational music therapy, music listening, music stimulation, or planned interventions delivered by therapists, family members, or researchers.

Control Conditions: Usual care, other non-musical stimulation, blank control, or other active treatments.

C (Study Types):

Design: RCTs or parallel-group-controlled studies.

Publication Type: Journal articles and dissertations published in Chinese or English with the full text available.

O (Outcome Measures):

Changes in the level of consciousness. Outcomes must include assessments using standardized scales such as changes in Coma Recovery Scale-Revised scores, changes in Glasgow Coma Scale scores, or consciousness recovery rates (e.g., transition from VS/UWS to MCS or emergence from MCS).

### 2.4 Exclusion criteria

Population: Non-human (animal) studies, patients without DoC (e.g., dementia, schizophrenia, or depression), and patients with locked-in syndrome.

Interventions: Interventions that were not primarily music-based (e.g., music used merely as uncontrolled background sound) or combined interventions (e.g., music therapy combined with medications), unless the experimental and control groups were identical in all other interventions besides music therapy, which would make it impossible to isolate the specific effects of music.

Study Types: Non-controlled studies (e.g., case reports, case series, reviews, commentaries, conference abstracts, and protocols) and studies for which the full text could not be obtained, or data were incomplete, even after contacting the original authors.

Outcomes: Studies that did not report extractable quantitative data related to the level of consciousness.

Duplicate Publications: For studies with duplicate publications, only the most complete or most recent report was included.

### 2.5 Data collection and analysis

Four authors (JYG, WL, SQZ, and CJL) participated in the screening process. Two authors (SQZ and CJL) removed duplicate records. Two authors (JYG and CJL) independently screened the literature based on the predefined inclusion and exclusion criteria. Titles and abstracts were first reviewed to exclude irrelevant studies, followed by a full-text review of potentially eligible studies. Any discrepancies regarding study inclusion were resolved through adjudication by a third author.

The study characteristics table extracted the following information: country of publication, study design, sample size, disease type, intervention method, intervention dosage, and outcome measures, as detailed in the Section 3.

### 2.6 Quality assessment and bias identification

The quality of the included RCTs and the risk of bias for each study were assessed using the Cochrane RoB 2 tool (Flemyng et al., 2023) across six domains: the randomization process, deviations from intended interventions, missing outcome data, measurement of the outcome, selection of the reported result, and overall bias. The quality of the included quasi-experimental studies and the risk of bias for each study were assessed using the ROBINS-I tool (Iheozor-Ejiofor et al., 2026) across eight domains: bias due to confounding, bias in selection of participants into the study, bias in classification of interventions, bias due to deviations from intended interventions, bias due to missing data, bias in measurement of outcomes, bias in selection of the reported result and overall bias. Quality control and bias assessment were conducted independently by two authors (JYG and CJL). Disagreements were resolved through discussion to reach a consensus. The quality evaluation form for the study is presented in Attachment 2.

### 2.7 Data and result extraction

For the multi-arm trials by (Puggina and da Silva 2015) and (Xiao et al. 2023)—each including three arms—we selected only the comparison that aligned with our definition of music as the core intervention. Specifically, from (Puggina and da Silva 2015), we compared the musical stimulation arm with the audio recording of “silence” arm, excluding the message stimulation arm. From (Xiao et al. 2023), we compared the live music therapy arm with the standard care arm, excluding the familial auditory stimulation arm. Data extraction was performed independently by two authors (JYG and CJL), who reviewed the literature and extracted relevant data on study outcome measures. The extracted data were verified by another author (WL) and imported into Review Manager V5.4.1 software (Copenhagen: The Nordic Cochrane Center, The Cochrane Collaboration, 2012) for meta-analysis.

### 2.8 Outcomes

Scores on consciousness level assessment scales were included in the meta-analysis. Outcome measures were assessed using the Coma Recovery Scale-Revised (CRS-R) and Glasgow Coma Scale (GCS).

### 2.9 Statistical analysis

Owing to the use of different assessment tools, the standardized mean difference (SMD) was used as the effect size for continuous data. Results for continuous variables are expressed as the SMD of changes from baseline to endpoint with 95% confidence intervals (CI). All data were pooled for analysis using a random-effects model or fixed-effects model based on whether heterogeneity is statistically significant. The degree of heterogeneity among the included studies was assessed using the *I*^2^ statistic. Given the relatively small number of studies and the limited total sample size, the statistical power of the *I*^2^ test is limited. Therefore, a *P*-value < 0.01 was considered indicative of significant heterogeneity (Huedo-Medina et al., 2006). To further characterize the degree of heterogeneity, a classification system was applied, categorized as low (< 25%), moderate (< 50%), or high (>75%) (Higgins et al., 2003). When high heterogeneity was present, subgroup and sensitivity analyses were conducted to explore potential sources of heterogeneity and assess the robustness of pooled results. Publication bias was examined using Egger's test (statistical significance was defined as *P* < 0.05). Forest plots were generated to synthesize studies reporting the same outcome measures, and sensitivity analyses were performed for the outcome indicators. Owing to the limited number of included studies (n = 7), meta-regression to explore potential sources of heterogeneity was not feasible. Consequently, a narrative summary of study characteristics and findings was provided, supplemented by quantitative meta-analysis of pooled effect sizes and heterogeneity statistics.

## 3 Results

### 3.1 Study selection

**Figure 1:**
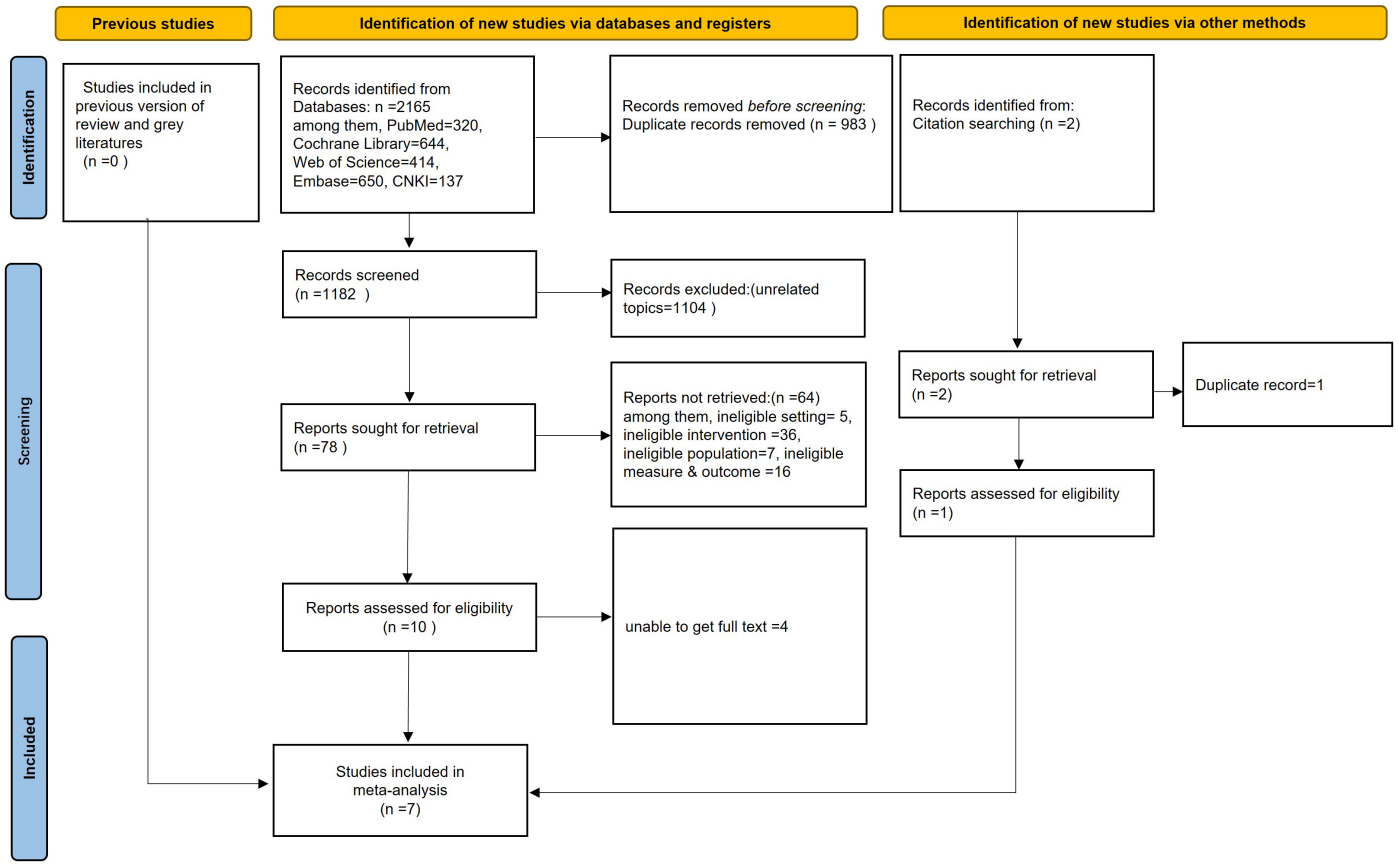
PRISMA flow diagram of the study selection procedure.

A total of 2165 studies were identified through database searches and Supplementary methods such as citation tracking. After three steps, removing duplicates, initial screening of titles and abstracts, and full-text review for eligibility, seven studies were included. The literature screening process is illustrated in Figure 1. Potentially eligible studies are listed in Attachment 3.

### 3.2 Study characteristics

Seven studies were included, comprising 296 patients, with 153 and 143 cases in the experimental and control groups, respectively. No significant differences in general characteristics between the two groups were reported across the included studies. Among these, four studies (Liang, 2008; Puggina and da Silva, 2015; Yekefallah et al., 2021; Xiao et al., 2023) were RCTs, and three studies (Sun and Chen, 2015; Sun et al., 2017; Gu et al., 2025) were quasi-experimental studies. The basic characteristics of the included studies are presented in Table 1.

**Table 1:** Characteristics of included studies (*N* = 7).

Items	Source	Research cycle (year)	Research site	Study design	Overall sample size	Group sample size	Lesion etiology	Age of participants (M ±SD, years)	Intervention	Intervention dose	Time points for evaluation	Outcomes
1	(Puggina and da Silva 2015)	2008.8–2009.9	Brazil	RCT	76	EG1: 30 EG2: 26 CG: 20	Disorders of consciousness	Total sample:42.5 ± 19 years	EG1: musical stimulation EG2: message stimulation CG: an audio recording of “silence” (ambient sound with no prior verbal instruction)	2–4 min per session, 2 sessions, 40 min apart; a total exposure of 4–8 min per condition	GCS was assessed before and 30–40 days after the intervention.	Glasgow Coma Scale (GCS) vital signs (heart rate, temperature, SaO_2_, blood pressure)
2	(Yekefallah et al. 2021)	2018–2019	Iran	RCT	54	EG:27 CG:27	Head trauma	EG:36.00 ± 13.37 years CG:39.51 ± 13.65 years	EG: musical stimulation (“Beach walk” music, between 60–80 decibels) CG: headphones were silent for 15 min without receiving any musical stimulation	15 min daily for 7 consecutive days	Daily before and after each session for 7 days	the Glasgow Coma Scale (GCS) the Richmond Agitation-Sedation Scale (RASS)
3	(Liang 2008)	2005.3–2007.9	China	RCT	60	EG:30 CG:30	Cerebral hemorrhage	EG:55.20 ± 8.70 years CG: 54.80 ± 7.90 years	EG: put on headphones for the patient to listen to music, and have a family member call his/her name CG: conventional treatment	About 20 min each time, 6 times/d, for 56d	Before treatment, 1, 2, 3, and 4 weeks after treatment	Glasgow Coma Scale (GCS)
4	(Xiao et al. 2023)	2019.12–2022.11	China	RCT	15	EG1: 5 EG2: 5 CG: 5	Disorders of consciousness (Minimally conscious state)	EG1: 26.8 ± 11.2 years EG2: 50.8 ± 10.1 years CG: 38.8 ± 15.15 years	EG1: the live music therapy supported by a music therapist EG2: familial auditory stimulation supported by patients' family members CG: standard care group, no auditory stimulation	30 min per session, five times per week for 4 weeks (20 sessions total)	Baseline and after 4 weeks of intervention	Glasgow Coma Scale (GCS) functional MRI-Diffusion Tensor Imaging (fMRI-DTI)
5	(Sun and Chen 2015)	2012.12–2013.12	China	Quasi-experimental study	40	EG:20 CG:20	Traumatic brain injury coma	EG: 39.35 ± 10.23 years CG: 40.05 ± 10.05 years	EG: music stimulation, patient's favorite and familiar music provided by family, played with plug-type earphones at 60-70 dB volume using an MP3 player. CG: no formal music therapy; patients received only routine medical treatment and care in the ICU	15-30 min per session, played every morning, afternoon, and before sleep at night (3 sessions per day), continuously for 4 weeks	Baseline (within 2 weeks of admission) and after 1 month of treatment	Glasgow Coma Scale (GCS) Quantitative EEG (QEEG) ratio (δ + θ)/(α + β)
6	(Gu et al. 2025)	2024.3–2025.3	China	Quasi-experimental study	42	EG:21 CG:21	Disorders of consciousness	EG: 52.76 ± 18.21 years CG: 49.86 ± 18.45 years	EG: MSOT along with conventional treatment. CG: conventional treatment for DoC and watched videos of family/friends' activities and short videos	30 min per session, five times per week for 8 weeks	Conducted twice a day for 5 consecutive days in week 1 (T0), week 5 (T1), and week 8 (T2) of the intervention. The best score from each assessment period was used.	Coma Recovery Scale-Revised (CRS-R)
7	(Sun et al. 2017)	2016.1–2016.12	China	Quasi-experimental study	40	EG:20 CG:20	Traumatic brain injury coma	EG: 43.60 ± 17.69 years CG: 50.10 ± 15.49 years	EG: music therapy with conventional treatment. CG: conventional treatment.	30 min per session, 3 times daily for 4 weeks	Baseline (pre-treatment) and at weeks 1, 2, 3, 4 of treatment	Glasgow Coma Scale (GCS) Coma Recovery Scale-Revised (CRS-R)

For multi-arm trials (Puggina and da Silva, 2015; Xiao et al., 2023), the overall sample size refers to the total number of enrolled participants; the number of participants included in the meta-analysis was 50 (music stimulation: 30, control: 20) and 10 (live music therapy: 5, control: 5), respectively. Details are provided in the Section 2.7.

EG, experimental group; CG, comparison/control group; RCT, randomized controlled trial; Quasi-experimental, non-randomized controlled trial; M, mean; SD, standard deviation; GCS, Glasgow Coma Scale; CRS-R, Coma Recovery Scale-Revised; MSOT, musical sensory orientation training.

### 3.3 Literature quality evaluation

**Figure 2:**
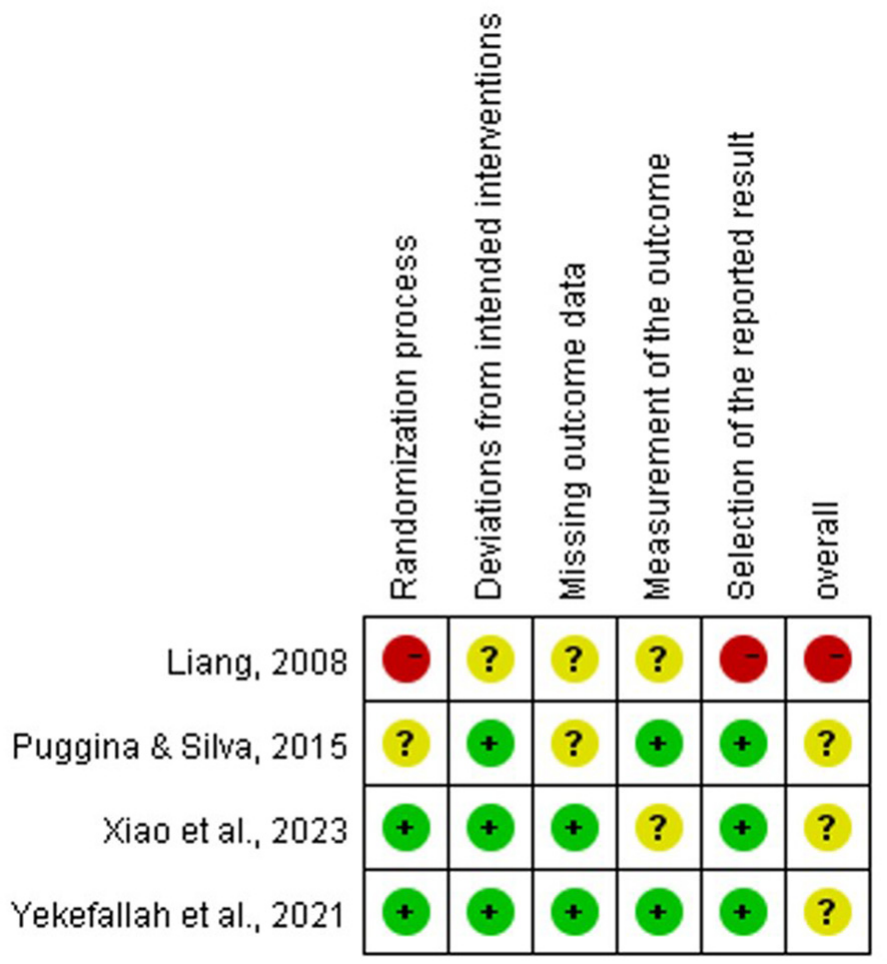
Risk of bias in included randomized controlled trial studies.

**Figure 3:**
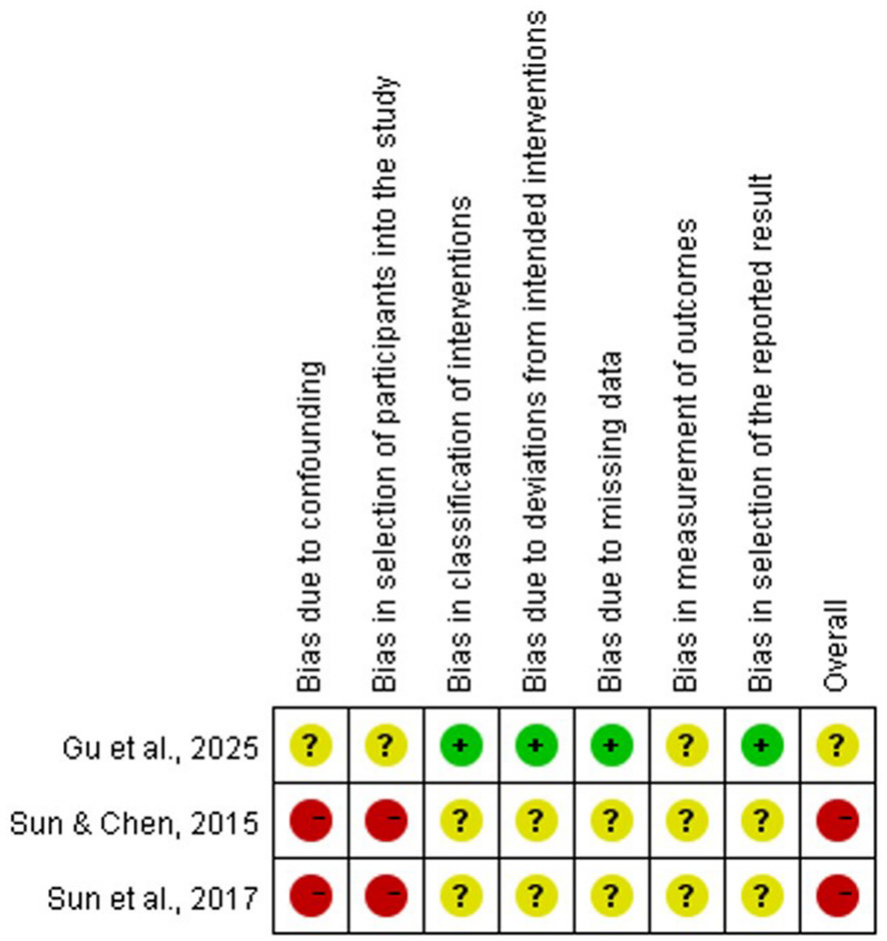
Risk of bias in included non-randomized controlled studies.

Of the seven studies included in this review, four RCTs were assessed for quality using the Cochrane RoB 2 tool, and three NRS were assessed using the ROBINS-I tool. The results are presented in Figures 2, 3.

### 3.4 Effectiveness of music therapy on the level of consciousness

**Figure 4:**
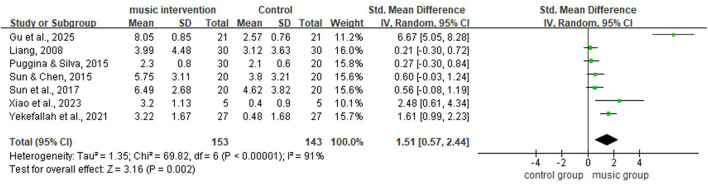
Forest plot for meta-analysis of all studies (random-effects model).

This meta-analysis used a random-effects model to evaluate changes from baseline to post-intervention in the music therapy group compared with the control group across the seven included studies. The analysis showed that the music therapy group achieved significantly higher scores for DoC (SMD = 1.51, 95% CI = [0.57; 2.44], *P* < 0.01; Figure 4). Given the heterogeneity observed among the studies, sensitivity and subgroup analyses were conducted to identify potential sources of heterogeneity.

### 3.5 Sensitivity analysis

**Figure 5:**
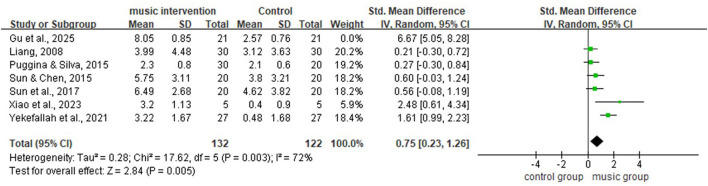
Forest plot of meta-analysis, excluding (Gu et al. 2025) (random-effects model).

**Figure 6:**
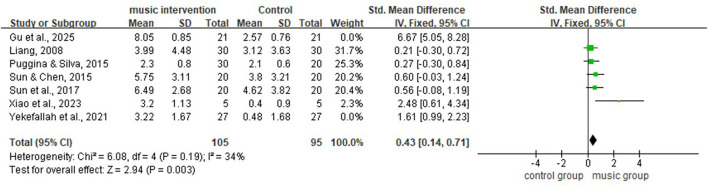
Forest plot of meta-analysis, excluding (Gu et al. 2025) and (Yekefallah et al. 2021) (fixed-effects model).

**Figure 7:**
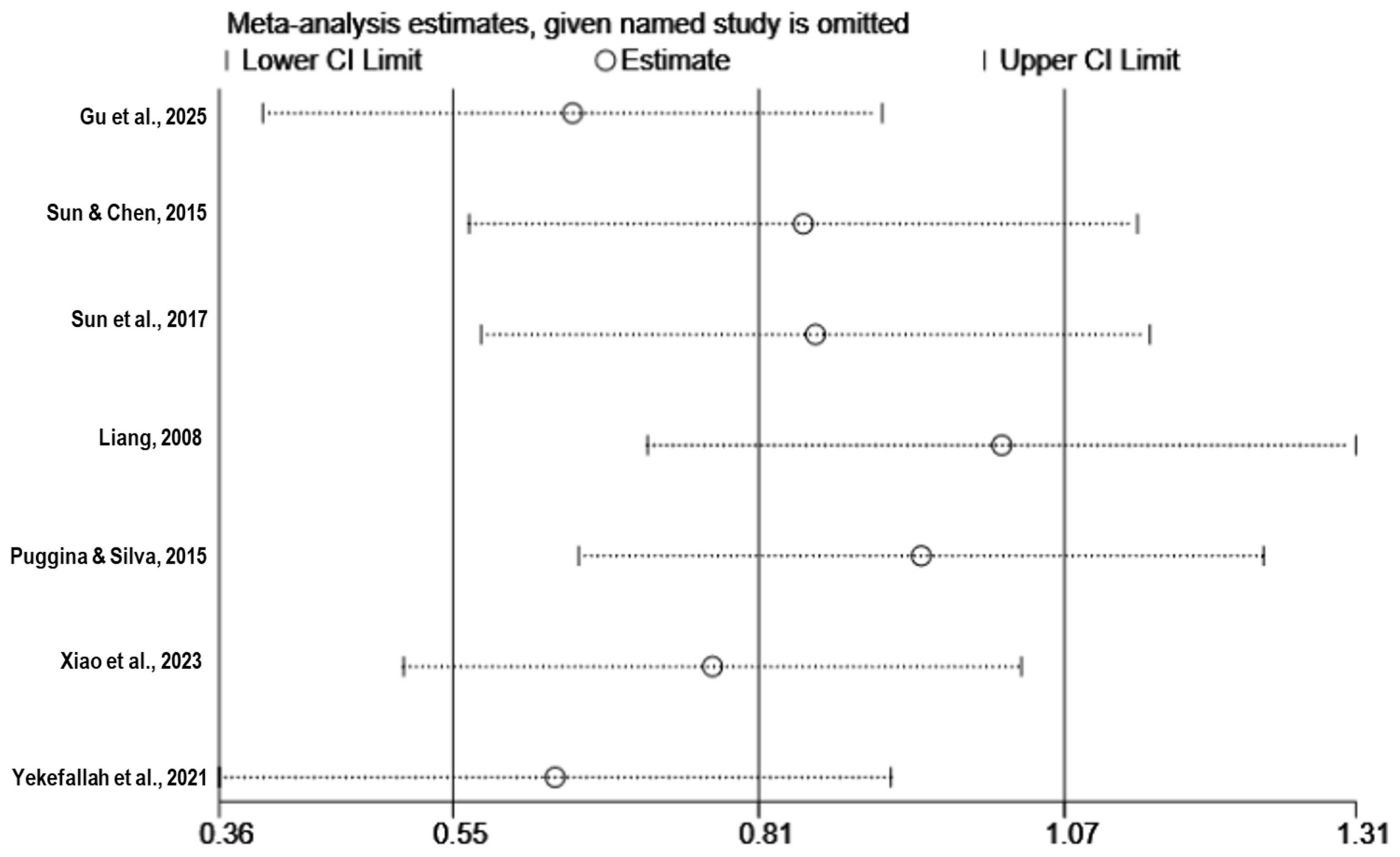
Meta-analysis estimates with each named study omitted in turn for all included studies.

Figure 4 shows substantial heterogeneity among the seven included studies (*I*^2^ = 91%, *P* < 0.01). A leave-one-out sensitivity analysis was performed to explore potential sources of heterogeneity (Table 2). The results indicated that after excluding the study by (Gu et al. 2025), heterogeneity decreased to a moderate level (*I*^2^ = 72%, *P* < 0.01; Figure 5). This study employed live interactive music therapy, which differs substantially from the passive music listening interventions used in the other included studies. This difference likely explains the marked reduction in heterogeneity after exclusion. Following the exclusion of this study, the pooled effect size of the remaining seven studies remained statistically significant (SMD = 0.75, 95% CI: 0.23–1.26, *P* < 0.01; Figure 5). After further excluding the study by (Yekefallah et al. 2021), the results showed no significant heterogeneity (*I*^2^ = 34%, *P* = 0.19; Figure 6), and the pooled effect size calculated using a fixed-effect model remained significant (SMD = 0.43, 95% CI: 0.14–0.71, *P* < 0.01; Figure 6). Furthermore, a sensitivity analysis plot generated using Stata/MP 17.0 software for all studies (Figure 7) showed that the direction of the pooled effect size did not reverse after sequentially omitting any single study, suggesting that the meta-analysis results were relatively robust.

**Table 2:** Leave-one-out sensitivity analysis: pooled effect estimates after omitting each study (based on 7 studies).

Study omitted	Effect size of omitted study	Weight in total synthesis (%)	Heterogeneity			Effect	
	SMD, 95% CI		Chi^2^	*P*	*I*^2^ (%)	*Z*	*P*
(Liang 2008)	0.21 [−0.30, 0.72]	16.0	63.14	< 0.001	92	3.11	< 0.01
(Puggina and da Silva 2015)	0.27 [−0.30, 0.84]	15.8	65.82	< 0.001	92	3.10	< 0.01
(Xiao et al. 2023)	2.48 [0.61, 4.34]	10.1	66.62	< 0.001	92	2.79	< 0.01
(Yekefallah et al. 2021)	1.61 [0.99, 2.23]	15.7	61.64	< 0.001	92	2.77	< 0.01
(Gu et al. 2025)	6.67 [5.05, 8.28]	11.2	17.62	=0.003	72	2.84	< 0.01
(Sun and Chen 2015)	0.60 [−0.03, 1.24]	15.6	69.44	< 0.001	93	2.99	< 0.01
(Sun et al. 2017)	0.56 [−0.08, 1.19]	15.6	69.20	< 0.001	93	3.01	< 0.01
All 7 studies (none omitted)	1.51 [0.57, 2.44]	100	69.82	< 0.001	91	3.16	< 0.01

(a) The column “Effect size of omitted study (SMD, 95% CI)” shows the standardized mean difference (SMD) and its 95% confidence interval for the study that was omitted in each row. (b) The column “Weight of omitted study (%)” indicates the weight that the omitted study contributed to the overall meta-analysis (based on the random-effects model). (c)All other columns (Chi^2^, P, I^2^, Z, P) present the heterogeneity and overall effect statistics after removing the study listed in the first column. d) For the row “All 7 studies (none omitted),” the effect size and weight refer to the pooled estimate of all seven studies

### 3.6 Subgroup analysis

**Figure 8:**
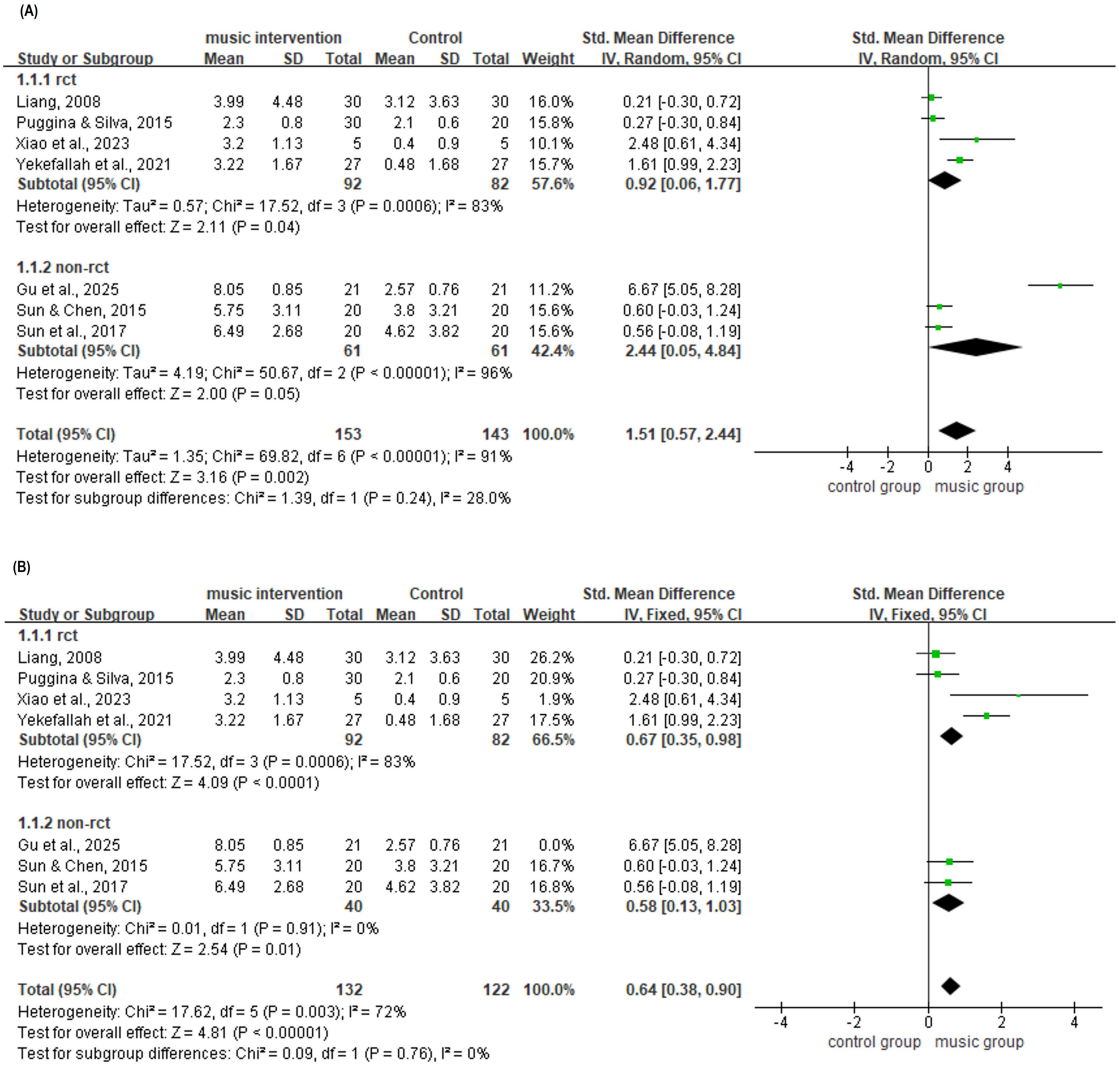
**(A)** Subgroup analysis of consciousness level by study type (random-effects model). **(B)** Subgroup analysis of consciousness level by study type, excluding (Gu et al. 2025) (fixed-effects model).

To further investigate the sources of heterogeneity, a subgroup analysis was conducted based on study design, categorizing studies into two subgroups: RCTs and NRS (Figure 8A). Heterogeneity analysis revealed varying degrees of heterogeneity within both subgroups. The RCT subgroup, comprising four studies, showed significant heterogeneity within the group (*I*^2^ = 83%, *P* < 0.01), with a pooled effect size of SMD = 0.92 (95% CI: 0.06–1.77, *P* = 0.04). The non-randomized controlled trial subgroup, also comprising three studies, showed substantial heterogeneity within the group (*I*^2^ = 96%, *P* < 0.01), with a pooled effect size of SMD = 2.44 (95% CI: 0.05–4.84, *P* = 0.05).

Sensitivity analysis of the three studies in the non-randomized controlled trial subgroup indicated that the heterogeneity primarily originated from a single study (Gu et al., 2025). The effect size of this individual study was substantially higher than that of the other studies (SMD = 6.67, 95% CI: 5.05–8.28). After excluding this study, no significant heterogeneity was observed among the remaining two NRS (*I*^2^ = 0%, *P* = 0.91). At this point, the effect size pooled using a fixed-effect model was SMD = 0.58 (95% CI: 0.13–1.03, *P* = 0.01; Figure 8B), which did not reach statistical significance, suggesting instability in the results.

The heterogeneity within the RCT subgroup (*I*^2^ = 83%) persisted after leave-one-out sensitivity analyses, with *I*^2^ ranging from 63% to 87%. This finding suggests that heterogeneity within the RCT subgroup may stem from combined differences across multiple studies, including clinical heterogeneity in intervention protocols, patient populations, and outcome measurements, as well as methodological differences potentially arising from unclear reporting of blinding and allocation concealment in individual studies (e.g., Liang, 2008).

The difference in heterogeneity between the two subgroups was not statistically significant (*I*^2^ = 28%, *P* = 0.24), indicating that differences in study design did not contribute to the primary heterogeneity observed in this meta-analysis.

### 3.7 Certainty of evidence

**Figure 9:**
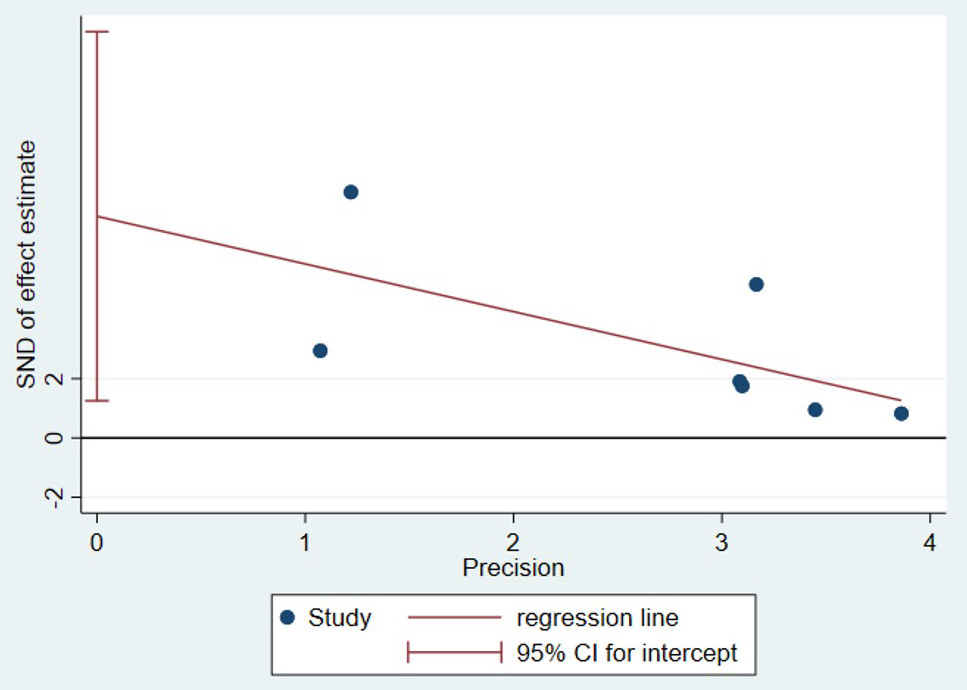
Egger's publication bias plot for all studies.

The quality of the evidence was assessed using the GRADE system, which includes five domains for downgrading: risk of bias, inconsistency, indirectness, imprecision, and publication bias, and three factors for upgrading: large effect size, dose-response gradient, and direction of plausible residual confounding. For the outcome of level of consciousness, the initial evidence quality was rated as low. Further, the Cochrane risk-of-bias tool was used to assess limitations in the included studies. Because the total weight proportion of studies rated as high risk exceeded 20%, the evidence was downgraded by one level in this domain. The pooled results showed an *I*^2^ greater than 50% (*P* < 0.01), and the heterogeneity could not be adequately explained through subgroup analyses; therefore, the evidence was downgraded by one level for inconsistency. Patient populations, interventions and outcome measures were generally consistent across the included studies; consequently, no downgrade was applied for indirectness. Regarding imprecision, the sample size was considered sufficiently large, and the CI did not cross the clinical decision threshold; therefore, no downgrade was applied for this domain. Publication bias was assessed using Egger's test, which yielded a *P*-value of 0.027 (< 0.05), indicating statistically significant publication bias and leading to a downgrade by one level. Figure 9 presents a publication bias plot constructed using Egger's linear regression method. For upgrading considerations, the pooled effect size was large (SMD = 1.51) and its 95% CI excluded the null value, meeting the GRADE criterion for a large effect; therefore, the evidence was upgraded by one level. After comprehensively considering both downgrading and upgrading factors, the overall quality of evidence was determined to be very low, as detailed in Table 3.

**Table 3:** Grade evaluation of evidence quality.

Certainty assessment							No. of patients		Effect		Certainty	Importance
No. of studies	Study design	Risk of bias	Inconsistency	Indirectness	Imprecision	Other considerations		placebo	Relative (95% CI)	Absolute (95% CI)		
7	Non-randomized trials	Serious^a^	Serious^b^	Not serious	Not serious	Serious^c^	153	143	–	SMD 1.51 SD more (0.57 more−2.44 more)	⊕○○○ Very low	Critical

Author(s).

Question: music intervention vs. other treatments.

Setting.

Bibliography: music intervention for consciousness disorders. Cochrane.

CI, confidence interval; SMD, standardized mean difference.

^a^According to the Cochrane risk assessment criteria, the weight of the literature with high-risk items was >20%.

^b^I^2^ > 50%, indicating heterogeneity.

^c^The Egger test indicates the presence of a statistically significant publication bias.

## 4 Discussion

This study conducted an exploratory meta-analysis on the awakening effects of music intervention in patients with DoC, including a total of seven controlled studies comprising four RCTs (Liang, 2008; Puggina and da Silva, 2015; Yekefallah et al., 2021; Xiao et al., 2023) and three non-randomized controlled studies (Sun and Chen, 2015; Sun et al., 2017; Gu et al., 2025), encompassing 296 patients with DoC. The pooled analysis results showed that the music intervention group demonstrated significantly greater improvement in consciousness level scores compared with the control group (SMD = 1.51, 95% CI: 0.57–2.44, *P* < 0.01), suggesting that music intervention may have a positive effect on awakening patients with DoC. However, this finding should be interpreted with caution in the context of substantial heterogeneity (*I*^2^ = 91%, *P* < 0.01).

### 4.1 Primary source of heterogeneity: differences in intervention modalities

Sensitivity analysis revealed that after excluding the study by (Gu et al. 2025), the overall heterogeneity decreased from 91% to 72% (moderate heterogeneity, *P* < 0.01), while the pooled effect size remained statistically significant (SMD = 0.75, 95% CI: 0.23–1.26, *P* = 0.005). Notably, following the exclusion of this study, heterogeneity among the remaining two NRS in the subgroup analysis decreased substantially from 96% to 0% (*P* = 0.91), and the effect size pooled using a fixed-effect model did not reach statistical significance (SMD = 0.58, 95% CI: 0.13–1.03, *P* = 0.01), further confirming the substantial impact of this study on the overall results.

This study employed live interactive music therapy, specifically Music Sensory Orientation Training (MSOT), in which therapists adjusted musical elements such as melody, rhythm, and volume in real time based on patients' respiratory rhythms and behavioral responses, while incorporating tasks involving visual tracking and auditory localization to promote consciousness recovery through multisensory integration. The intervention protocol consisted of 30-min sessions five times weekly for 8 weeks, representing one of the longest intervention durations and the highest levels of interactivity among the included studies.

In contrast, the remaining six studies utilized passive music listening, in which patients listened to pre-recorded or live-performed music (Liang, 2008; Sun and Chen, 2015; Puggina and da Silva, 2015; Sun et al., 2017; Yekefallah et al., 2021; Xiao et al., 2023). This finding suggests that intervention modality (interactive vs. passive) may represent an important source of heterogeneity. Interactive music therapy may activate broader neural networks, including the prefrontal cortex, cingulate gyrus, and auditory cortex, through real-time emotional and rhythmic interaction between therapist and patient, thereby generating more pronounced neurophysiological effects (Xiao et al., 2024). However, given that only one study employed this modality, its effect size substantially influenced the overall results, warranting further investigation for validation.

After further excluding the study by (Yekefallah et al. 2021), the overall heterogeneity decreased (*I*^2^ = 34%, *P* = 0.19), and the pooled effect size remained significant under the fixed-effect model (SMD = 0.43, 95% CI: 0.14–0.71, *P* < 0.01). This study's intervention protocol consisted of 15 min of passive music listening daily for consecutive days, using fixed light music (“Beach walk”) with a tempo of 60–80 beats per minute. Its characteristics included a relatively short intervention duration of 7 days, the shortest among the included studies, a single music type, and a lack of personalization based on patient preferences. All patients had DoC following traumatic brain injury (GCS scores 5–8). The substantial influence of this study on heterogeneity and pooled effect size may be attributed to its lower intervention intensity and shorter duration, which likely resulted in a relatively smaller effect size that exerted a diluting effect in the overall analysis. Following its exclusion, the effect sizes of the remaining studies became more consistent, with heterogeneity substantially reduced.

### 4.2 Heterogeneity in patient characteristics and outcome measures

Considerable variability existed in patient etiology across the included studies, encompassing traumatic brain injury (Sun and Chen, 2015; Sun et al., 2017; Yekefallah et al., 2021; Xiao et al., 2023), intracerebral hemorrhage (Liang, 2008) and DoC without specified subtypes (Puggina and da Silva, 2015; Gu et al., 2025). Different etiologies may influence patient responsiveness to music intervention. For instance, patients with traumatic brain injury may exhibit greater neuroplasticity, whereas patients with chronic cerebrovascular disease may demonstrate slower responses. Furthermore, discrepancies existed in the measurement tools and time points used for outcome assessment. Six studies employed the GCS (Liang, 2008; Sun and Chen, 2015; Puggina and da Silva, 2015; Sun et al., 2017; Yekefallah et al., 2021; Xiao et al., 2023), and two studies utilized the CRS-R (Sun et al., 2017; Gu et al., 2025). Assessment time points ranged from immediately post-intervention (Yekefallah et al., 2021) to 30–40 days following intervention (Puggina and da Silva, 2015). The GCS and CRS-R differ in sensitivity and specificity, with the CRS-R recognized as the gold standard for consciousness assessment in patients with DoC. However, its application was inconsistent across the included studies. These factors collectively may have contributed to the substantial heterogeneity observed among the studies.

### 4.3 Subgroup analysis: study design was not the primary source of heterogeneity

Subgroup analysis based on study design (RCT vs. NRS) revealed no statistically significant difference in heterogeneity between the two subgroups (*I*^2^ = 28%, *P* = 0.24), indicating that study design itself was not the primary source of heterogeneity in this meta-analysis. However, substantial heterogeneity persisted within the RCT subgroup (*I*^2^ = 83%, *P* = 0.0006), which could not be fully resolved through sensitivity analysis. Examination of the study characteristics table revealed considerable differences among the four RCTs in sample size, ranging from 10 to 50 patients, intervention protocols including music type, duration and frequency and patient populations including intracerebral hemorrhage, traumatic brain injury and DoC (Liang, 2008; Puggina and da Silva, 2015; Yekefallah et al., 2021; Xiao et al., 2023). For instance, (Liang 2008) employed music combined with family voices with an intervention duration extending to 8 weeks, whereas (Xiao et al. 2023) utilized live music performed by a music therapist but included an extremely small sample size of 5 cases per group. This clinical heterogeneity likely constituted the primary explanation for the heterogeneity observed within the RCT subgroup rather than methodological quality issues alone.

### 4.4 Comparison with previous reviews

The findings of this study are consistent with previous systematic reviews. Grimm and Kreutz, 2018, in their systematic review of quantitative studies, suggested that music intervention may improve behavioral scores in patients with DoC; however, they were unable to perform a meta-analysis (Grimm and Kreutz, 2018). Although the meta-analysis by (Li et al. 2020) conducted quantitative synthesis, the included studies comprised both RCTs and observational studies without stratified analysis by study design. A scoping review by (Lancioni et al. 2021) indicated that different types of music interventions may influence patient outcomes through distinct mechanisms. Building upon this foundation, the present study, based on the detailed characteristics of the seven included studies, identified intervention modality (interactive vs. passive) and intervention intensity, including duration and degree of personalization, as key sources of heterogeneity through sensitivity analysis, providing more refined hypotheses for future research.

### 4.5 Limitations

This study has some limitations. First, the number of included studies was limited, only seven, three of which were NRS (Gu et al., 2025; Sun and Chen, 2015; Sun et al., 2017), which may affect the strength of the evidence. Second, substantial heterogeneity was observed among the studies. Although potential sources were explored through sensitivity and subgroup analyses, the limited number of studies precluded quantitative analyses such as meta-regression. Third, sensitivity analyses revealed that the pooled results were sensitive to individual studies. Heterogeneity decreased after excluding (Gu et al. 2025) and decreased further after excluding (Yekefallah et al. 2021), while the effect remained significant. These findings indicate insufficient robustness of the current evidence and suggest that the overall conclusions may depend strongly on the characteristics of specific studies. Fourth, most studies had relatively small sample sizes, ranging from 10 to 50 patients, and publication bias was suggested (Egger's test *P* = 0.0027). Fifth, considerable variability existed in music intervention protocols, including type, duration, frequency, and degree of personalization, and in patient characteristics, including etiology, baseline consciousness level, and disease duration, which may limit the clinical generalizability of the findings. The GRADE rating classified the quality of evidence as “very low,” further emphasizing the preliminary nature of the conclusions.

### 4.6 Implications for future research

Based on the findings of this study and the characteristics of the included studies, future research should focus on the following aspects. First, large-scale, multicenter RCTs should be conducted, particularly with stratified evaluations of the effects of music intervention across different DoC subtypes (VS/UWS vs. MCS) and etiologies (traumatic vs. non-traumatic). Second, intervention modalities should be clearly distinguished and standardized. Interactive music therapy and passive music listening should be evaluated separately, and the optimal parameters for each, such as minimum effective duration, frequency, music type, and degree of personalization, should be explored. Third, studies should provide detailed reports of intervention protocols, including the rationale for music selection, whether personalized, therapist qualifications, and interactive content, to enable more refined subgroup analyses in future meta-analyses. Fourth, unified and validated outcome measurement tools should be adopted, with the CRS-R recommended, and assessment time points, for example, immediately post-intervention, 4 weeks, and 8 weeks, should be clearly specified while ensuring blinding of outcome assessors. Fifth, future meta-analyses should be pre-registered, and plans should include sensitivity analyses and meta-regression to explore sources of heterogeneity more systematically.

### 4.7 Conclusion

This exploratory meta-analysis provides preliminary evidence suggesting that music intervention may have positive effects on awakening patients with DoC, as reflected by improvements in consciousness-related behavioral scale scores. However, given the limited number of included studies, only seven, substantial heterogeneity among studies, and the quality of evidence downgraded to “very low” by GRADE, these findings should be interpreted with caution. Analysis of the characteristics of the seven included studies indicated that the observed heterogeneity stemmed primarily from differences in intervention modalities (interactive vs. passive music therapy) and intervention intensity, including duration and degree of personalization, rather than study design itself. In view of the clinical heterogeneity of the patient population and the diversity of intervention protocols, future research should prioritize large-scale, methodologically rigorous RCTs with stratified analyses by DoC subtype and intervention type, employing standardized intervention protocols, such as standardized operating procedures for MSOT, and outcome measures such as the CRS-R. Only through such high-quality studies can the therapeutic value of music intervention for patients with DoC be reliably established, thereby providing an evidence-based foundation for clinical practice.
