# Supplementary material for: Exploratory meta-analysis of the effect of music intervention on arousal promotion in patients with disorders of consciousness: evidence from controlled studies
Source: Front Neurosci. 2026 May 8;20:1831090. doi: 10.3389/fnins.2026.1831090 (PMC13195019; doi:10.3389/fnins.2026.1831090)

**Supplementary material 1 Search strategy**

**Supplementary grey literature search:**

ProQuest Dissertations & Theses Global: searched using (disorders of consciousness OR vegetative state OR minimally conscious state OR coma) AND (music therapy OR music intervention) – limited to English and Chinese, no date restriction.

Google Scholar: first 200 records sorted by relevance using the same keyword combination, manually screened for potentially eligible studies (due to limited export capabilities).

China National Knowledge Infrastructure (CNKI) already includes dissertations; no additional grey literature search was performed for Chinese studies.

Hand‑searching of reference lists of all included studies and relevant systematic reviews (Grimm & Kreutz, 2018; Li et al., 2020; Lancioni et al., 2021) to identify additional controlled studies not captured by electronic databases.

Contacting experts in the field of music therapy and disorders of consciousness to inquire about unpublished or ongoing studies (no additional studies were identified through this route).

The primary electronic database searches (detailed below) were conducted in October 2025. The search results from the broad strategy were screened independently by two reviewers. Non‑randomized studies meeting our inclusion criteria were retained if they had a concurrent control group (non‑randomized controlled trial or quasi‑experimental design).

**Database‑specific search strategies:**

| PubMed  (searched 3 Oct 2025) | 1# Search: ((((((((((((((((Disorders of Consciousness[MeSH Terms]) OR (Persistent Vegetative State[MeSH Terms])) OR (Consciousness[MeSH Terms])) OR (Consciousness Disorders[MeSH Terms])) OR (disorder of consciousness[Title/Abstract])) OR (disorders of consciousness[Title/Abstract])) OR (DOC[Title/Abstract])) OR (vegetative state[Title/Abstract])) OR (VS[Title/Abstract])) OR (unresponsive wakefulness syndrome[Title/Abstract])) OR (UWS[Title/Abstract])) OR (minimally conscious state[Title/Abstract])) OR (MCS[Title/Abstract])) OR (coma[Title/Abstract])) OR (comatose[Title/Abstract])) OR (consciousness recovery[Title/Abstract])) OR (arousal[Title/Abstract]) 1,307,703  2# Search: ((((((((Music Therapy[MeSH Terms]) OR (Music[MeSH Terms])) OR (music therap*[Title/Abstract])) OR (music intervention*[Title/Abstract])) OR (music stimulation[Title/Abstract])) OR (music exposure[Title/Abstract])) OR (auditory stimulation[Title/Abstract])) OR (melodic intonation therapy[Title/Abstract])) OR (music-based intervention*[Title/Abstract])  26,350  3# Search: #1 AND #2 1,448  4# Search:#1 AND #2 AND ((((randomized controlled trial[Publication Type]) OR (randomized[Title/Abstract])) OR (placebo[Title/Abstract]) OR (Non-randomized controlled trial) 320 |
| --- | --- |
| Cochrane Library  (searched 3 Oct 2025) | #1 MeSH descriptor: [Consciousness Disorders] explode all trees 1 479  #2 MeSH descriptor: [Persistent Vegetative State] explode all trees 94  #3 MeSH descriptor: [Consciousness] explode all trees 556  #4 #1 OR #2 OR #3 1 965  #5 (disorder of consciousness):ti,ab,kw OR (disorders of consciousness):ti,ab,kw OR (DOC):ti,ab,kw OR (vegetative state):ti,ab,kw OR (VS):ti,ab  ,kw OR (unresponsive wakefulness syndrome):ti,ab,kw OR (UWS):ti,ab,kw OR (minimally conscious state):ti,ab,kw OR (MCS):ti,ab,kw OR (coma):ti,ab,kw OR (comatose):ti,ab,kw OR (consciousness recovery):ti,ab,kw OR (arousal):ti,ab,kw 333,199  #6 #4 OR #5 334,157  #7 MeSH descriptor: [Music Therapy] explode all trees 1,541  #8 MeSH descriptor: [Music] explode all trees 1,327  #9 #7 OR #8 2,500  #10 (music therap*):ti,ab,kw OR (music intervention*):ti,ab,kw OR (music stimulation):ti,ab,kw OR (music exposure):ti,ab,kw OR (auditory stimulation):ti,ab,kw OR (melodic intonation therapy):ti,ab,kw OR (music-based intervention*):ti,ab,kw 11,125  #11 #9 OR #10 11,467  #12 #6 AND #11 1,279  #13 #11 AND #12 1,278  #14 #13 AND Non-randomized controlled trial 4  #15 #13 AND randomized controlled trial 710  #16 #14 OR #15 714  As of Oct 3, 2025 644 |
| Web of Science  (searched 3 Oct 2025) | #1  Disorders of Consciousness or Persistent Vegetative State or Consciousness or Consciousness Disorders or disorder of consciousness or disorders of consciousness or DOC or vegetative state or VS or unresponsive wakefulness syndrome or UWS or minimally conscious state or MCS or coma or comatose or consciousness recovery or arousal 1 607 261  #2  Music Therapy or Music or music therap* or music intervention* or music stimulation or music exposure or auditory stimulation or melodic intonation therapy or music-based intervention* 187 412  3# Non-randomized controlled trial or randomized controlled trial 912 335  #1 AND #2 AND #3 414 |
| Embase  (searched 3 Oct 2025) | #1  'consciousness'/exp OR 'persistent vegetative state':ab,ti OR 'consciousness':ab,ti OR 'consciousness disorders':ab,ti OR 'disorder of consciousness':ab,ti OR 'disorders of consciousness':ab,ti OR 'doc':ab,ti OR 'vegetative state':ab,ti OR 'vs':ab,ti OR 'unresponsive wakefulness syndrome':ab,ti OR 'uws':ab,ti OR 'minimally conscious state':ab,ti OR 'mcs':ab,ti OR 'coma':ab,ti OR 'comatose':ab,ti OR 'consciousness recovery':ab,ti OR 'arousal':ab,ti 2 462 874  #2  'music therapy'/exp OR 'music':ab,ti OR 'music therap*':ab,ti OR 'music intervention*':ab,ti OR 'music stimulation':ab,ti OR 'music exposure':ab,ti OR 'auditory stimulation':ab,ti OR 'melodic intonation therapy':ab,ti OR 'music-based intervention*':ab,ti 40 792  3# Non-randomized controlled trial or randomized controlled trial 1 589 477  #1 AND #2 AND #3 650 |
| CNKI  (searched 3 Oct 2025) | ( 音乐治疗 ) + ( 音乐疗法 + 音乐干预 + 音乐刺激 + 音乐暴露 + 听觉刺激 + 旋律发音治疗 + 基于音乐干预 ) AND ( 意识障碍 ) + ( 持续植物状态 + 植物状态 + 无反应性觉醒综合征 + 微意识状态 + 昏迷 + 昏迷状态 + 意识恢复 + 觉醒训练 ) 137 |


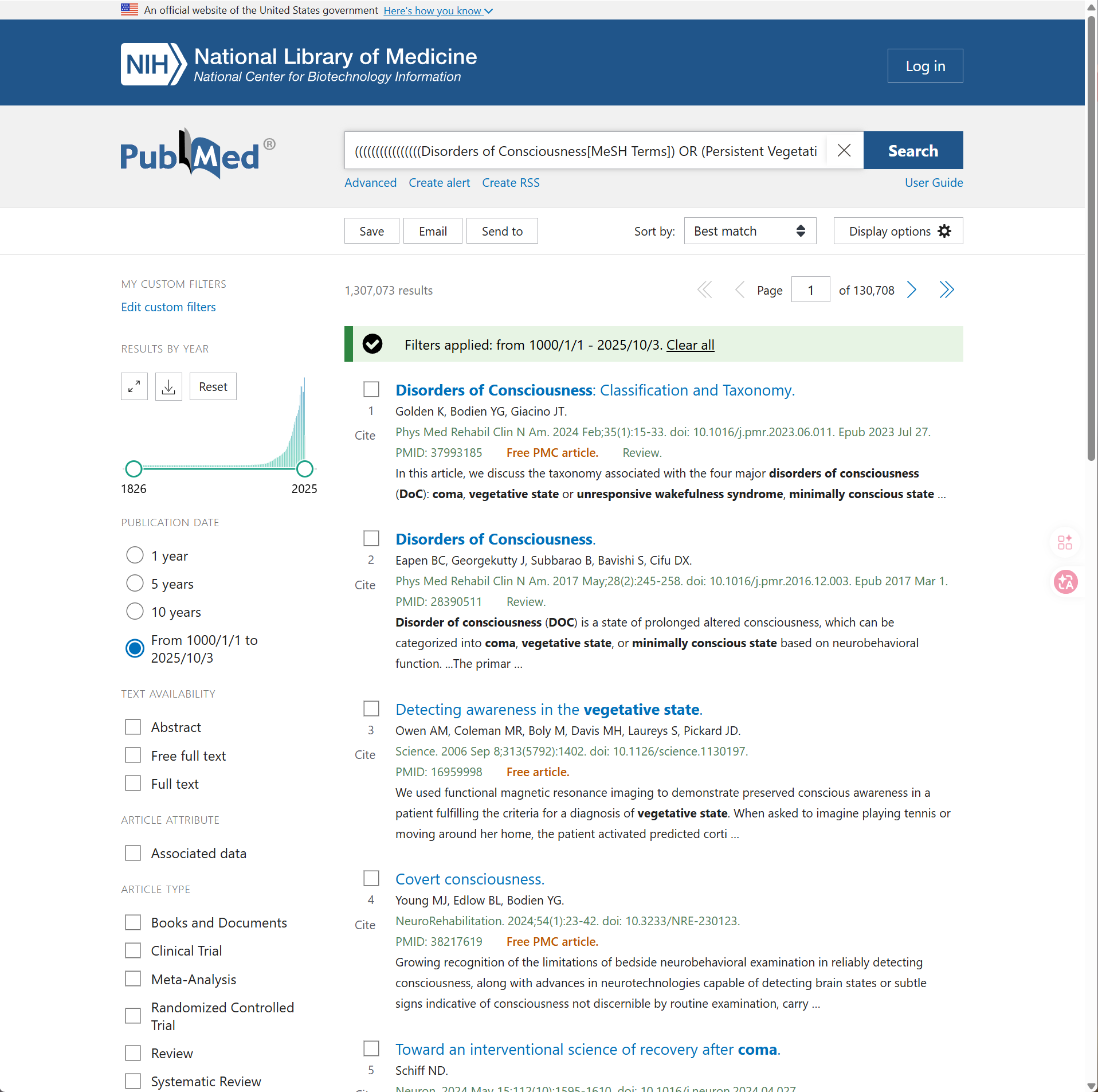


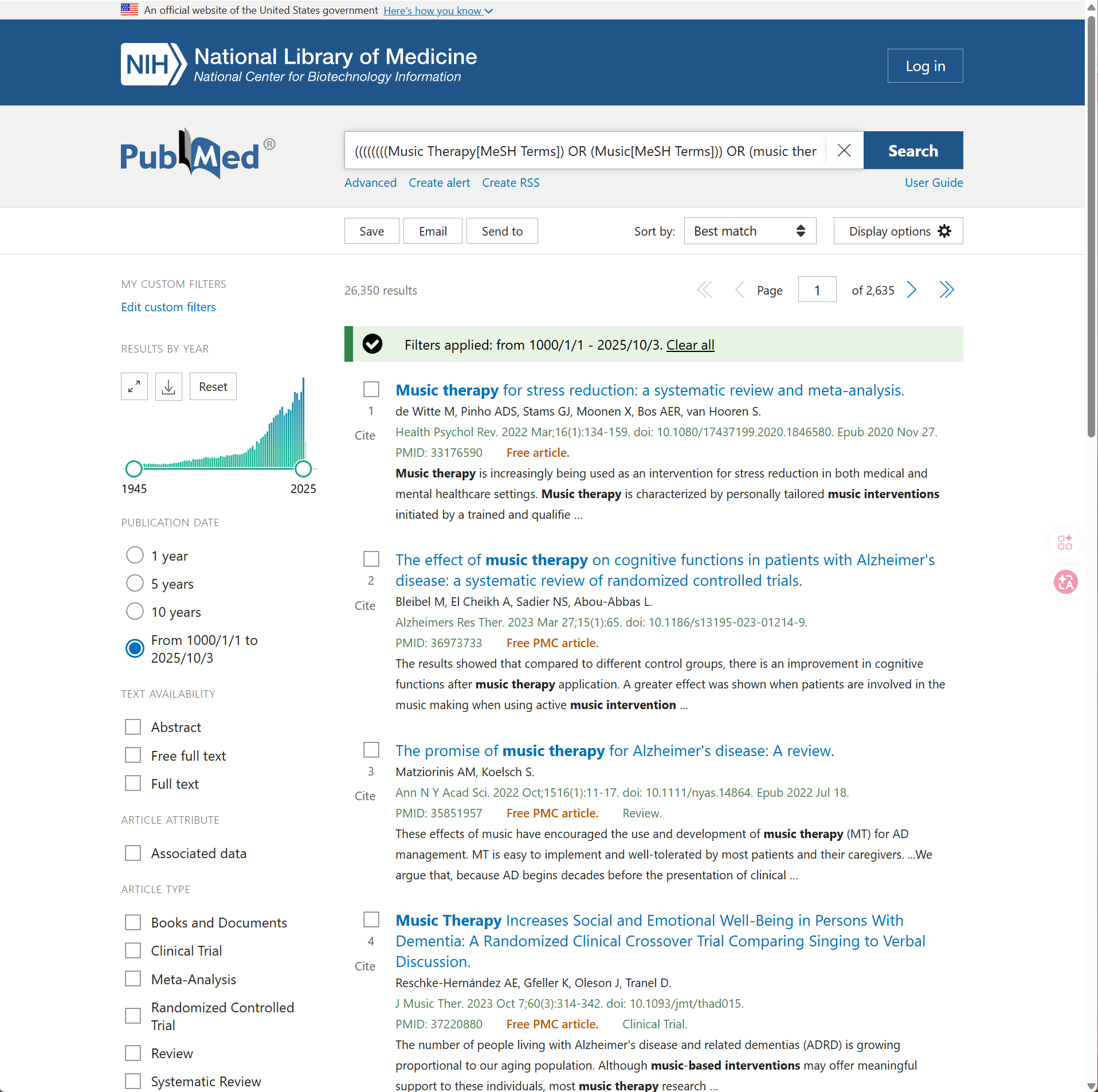


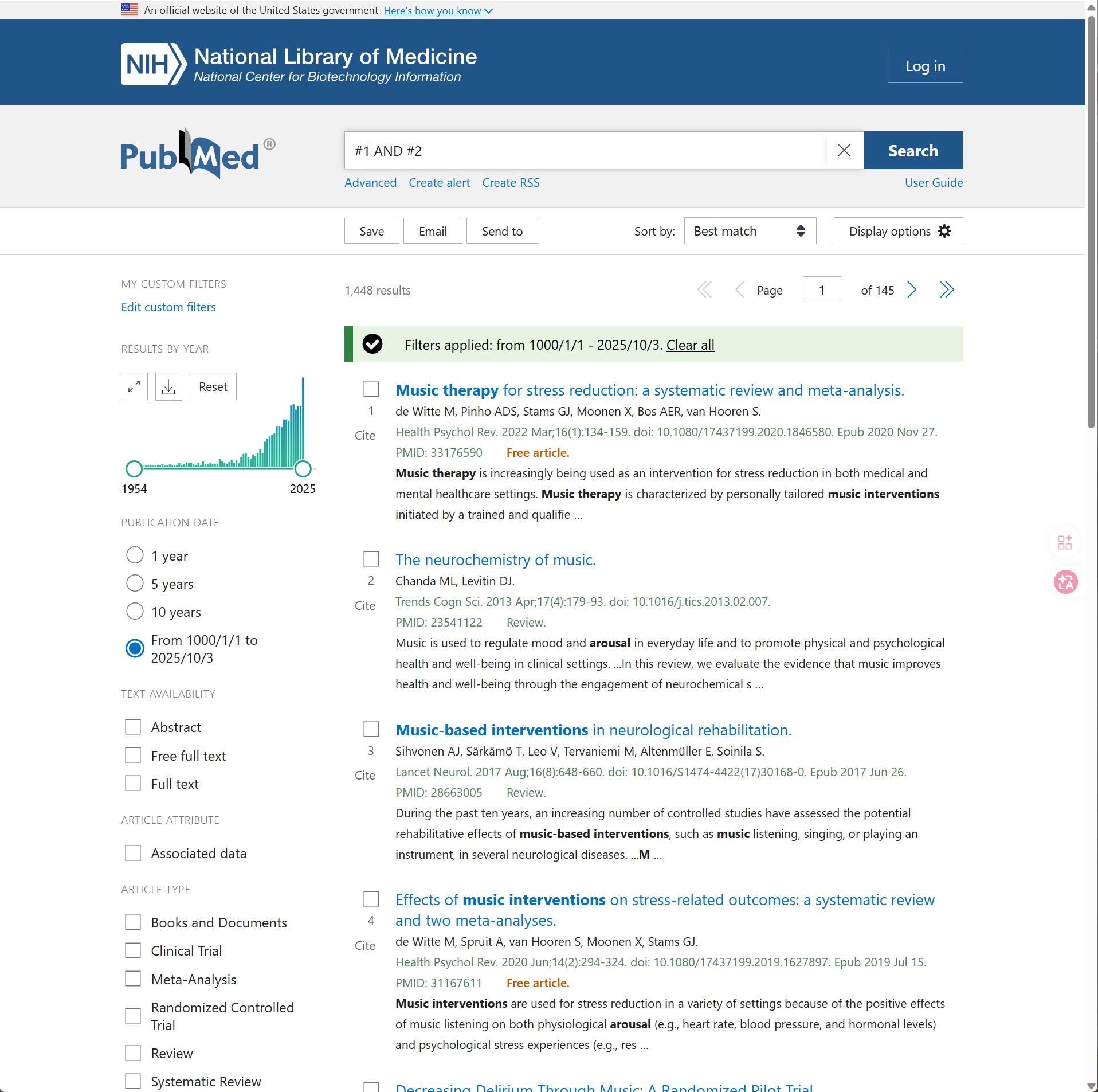


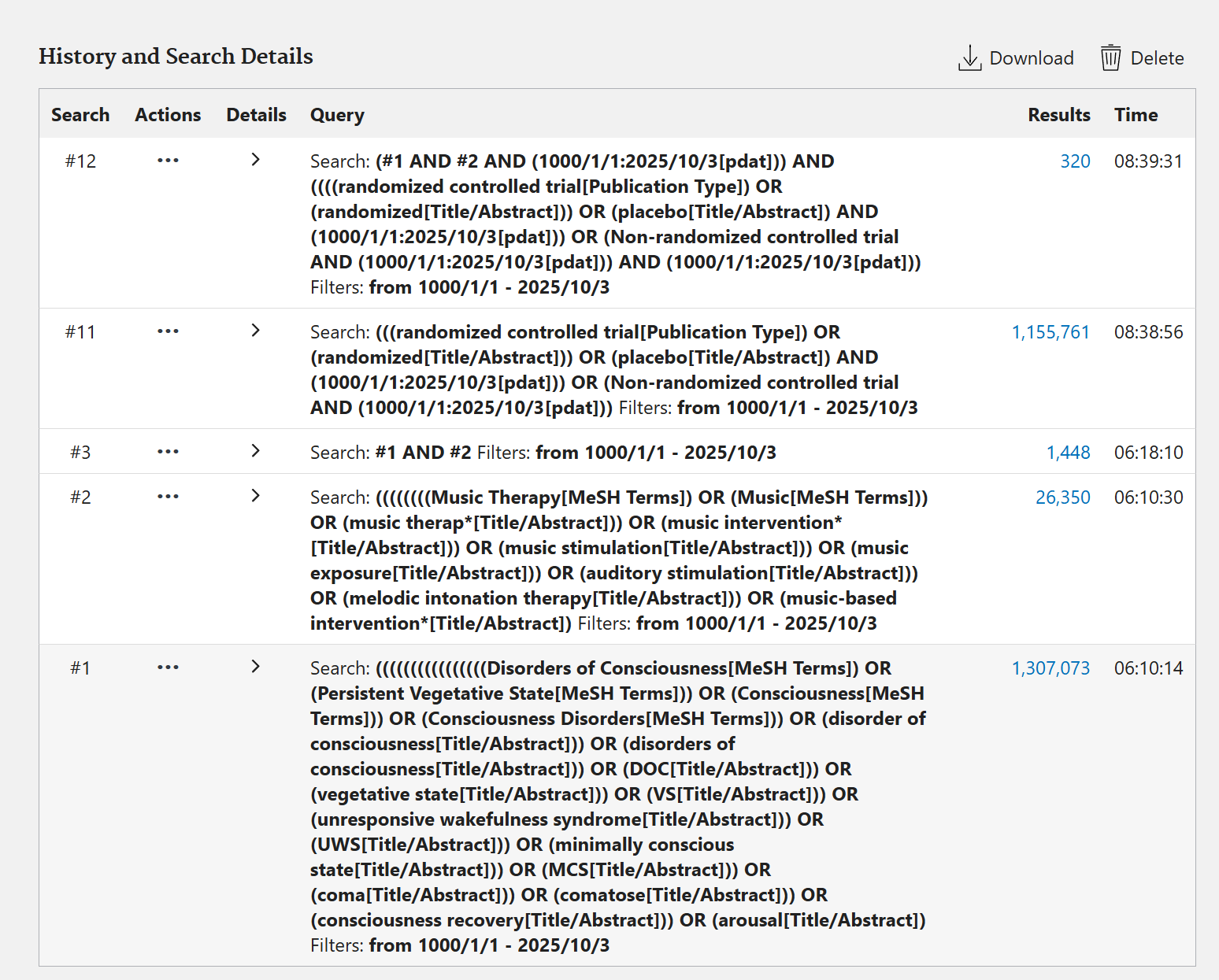


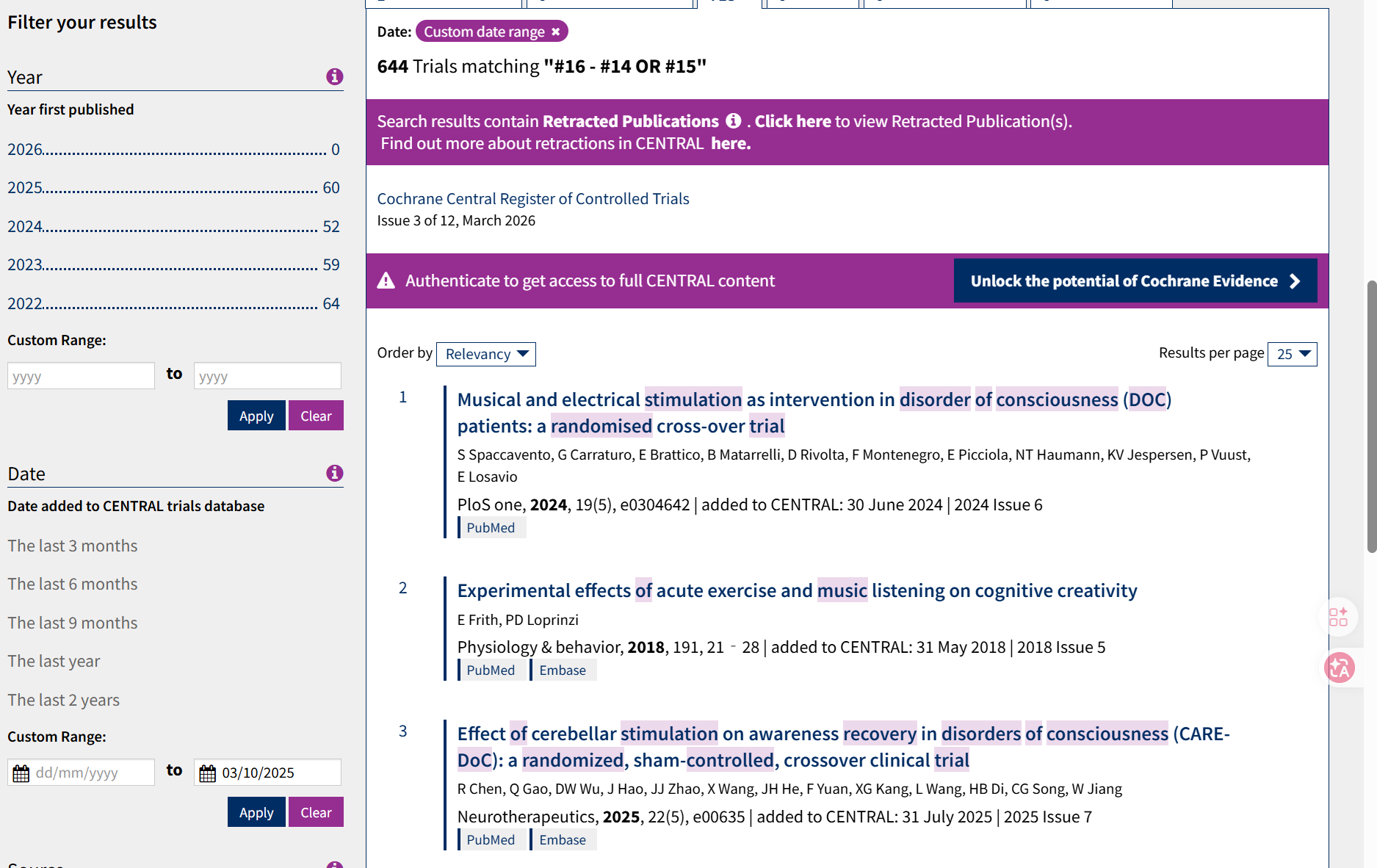


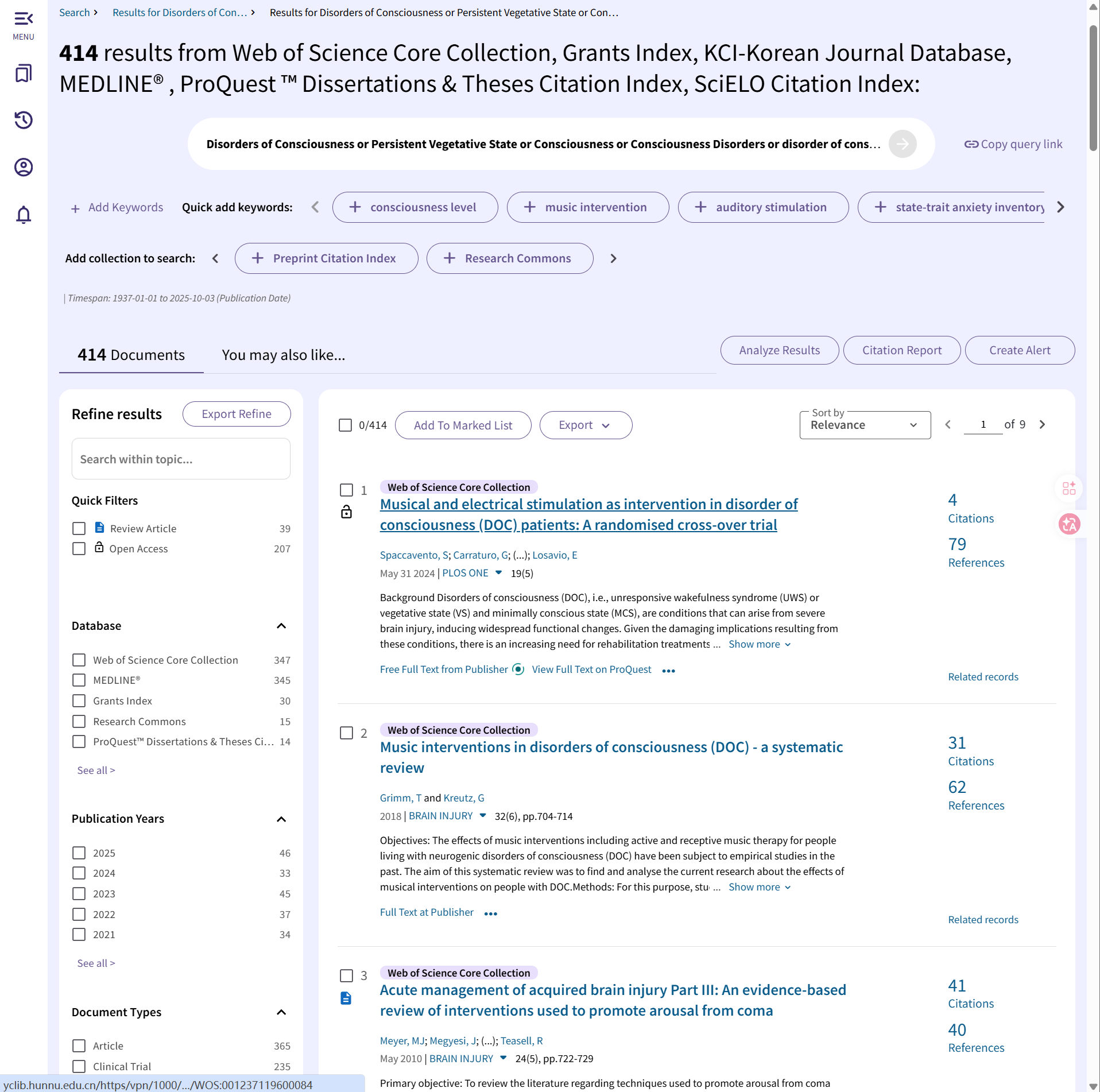


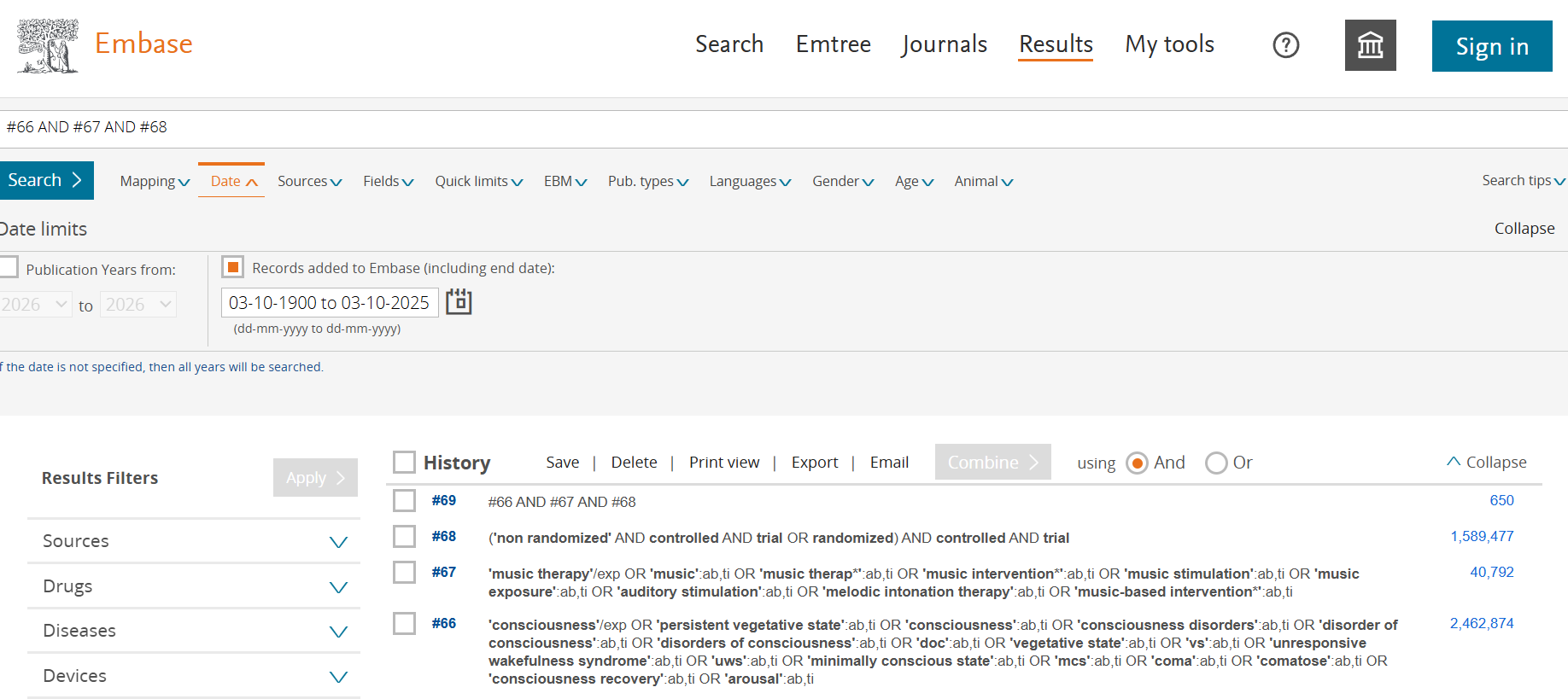


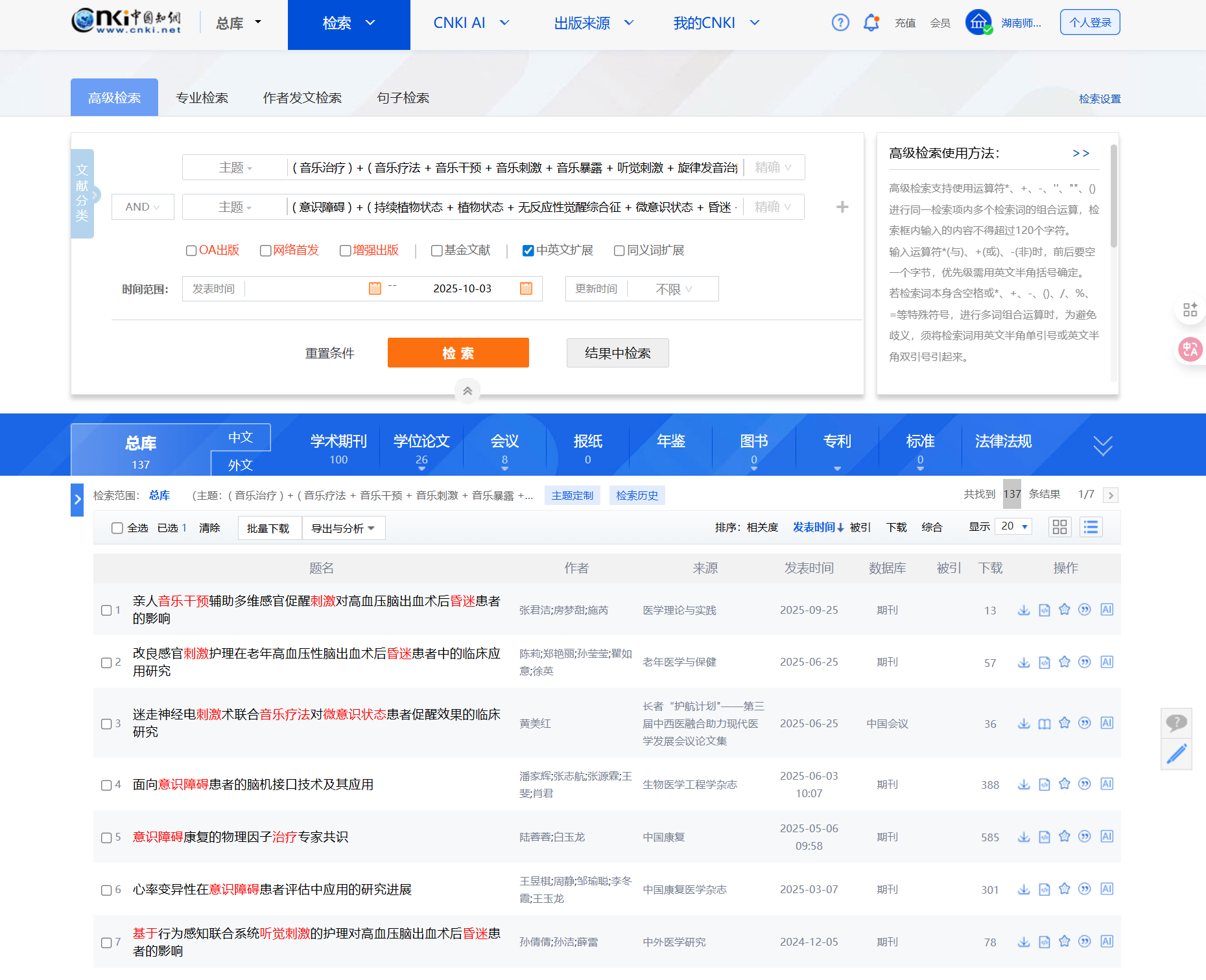

Supplement: Supplementary file 1 [file Data_sheet_1.docx]
