# Supplementary material for: Exploratory meta-analysis of the effect of music intervention on arousal promotion in patients with disorders of consciousness: evidence from controlled studies
Source: Front Neurosci. 2026 May 8;20:1831090. doi: 10.3389/fnins.2026.1831090 (PMC13195019; doi:10.3389/fnins.2026.1831090)
Supplement: Supplementary file 3 [file Data_Sheet_3.docx]

**Supplementary material 3**

**The list of studies that were excluded after refined screening**

**Ineligible setting (n=5)**

| # | Reference | Exclusion reason |
| --- | --- | --- |
| 1 | Bender, A., et al. (2023). The Neurological Rehabilitation of Adults With Coma and Disorders of Consciousness. *Dtsch Arztebl Int*, 120(37), 605-612. | **Guideline, not original study.** |
| 2 | Şirin Gök, M., & Balci Akpinar, R. (2025). The effect of different auditory stimuli on vital signs and consciousness level in intensive care patients: Music, nature-based sound and voices of patients' relatives. *Nurs. Open*, 12, e70273. | **Cross-over (within-subject) design; incompatible with parallel-group meta-analysis.** |
| 3 | Yan, J., et al. (2024). Combining HD-tDCS with music stimulation for patients with prolonged disorders of consciousness: Study protocol for an RCT trial. *NeuroRehabilitation*, 54(3), 495-504. | **Protocol only, no results.** |
| 4 | Purushe, D., et al. (2023). Effectiveness of sensory stimulation and early mobility on consciousness, mental state, RLA stage, and hospital stay in patients with Traumatic brain injury: A research protocol. *F1000Research*, 12, 1427. | **Protocol only, no results.** |
| 5 | 姚舜, 唐雅彬, 李水艳, 任欣然, 谢秋幼, 等. (2023). 音乐治疗辅助意识障碍患者康复的研究进展. *中国康复医学杂志*, 38(3), 427-431. | **Review, not original study.** |

**Ineligible intervention (n=36)**

*Note: Studies where the intervention was not primarily music-based, or where music was combined with other active interventions that could not be isolated.*

| # | Reference | Exclusion reason |
| --- | --- | --- |
| 1 | Mohammadi, M. K., et al. (2019). The effects of familiar voices on the level of consciousness among comatose patients. *J. Pharm. Res. Int.*, 27(2), 1-8. | **Intervention = familiar voice (not music).** |
| 2 | Khojeh, A., et al. (2018). The effect of organized auditory stimulation with a familiar voice on pain intensity and physiological indices of comatose patients. *J. Res. Med. Dent. Sci.*, 6(3), 69-77. | **Intervention = familiar voice (not music).** |
| 3 | Froutan, R., et al. (2020). The effect of music therapy on physiological parameters of patients with traumatic brain injury. *Complement. Ther. Clin. Pract.*, 40, 101216. | **Music combined with family recollection (cannot isolate music effect).** |
| 4 | Park, S., & Davis, A. E. (2016). Effectiveness of direct and non-direct auditory stimulation on coma arousal after traumatic brain injury. *Int. J. Nurs. Pract.*, 22(4), 391-396. | **Intervention includes direct voice and music; cross-over design.** |
| 5 | Hoseini, S. H., et al. (2022). Effectiveness of auditory sensory stimulation on level of consciousness and cognitive function in traumatic brain injury patients. *Nurs. Pract. Today*, 9(4), 349-359. | **Intervention = auditory sensory stimulation (not primarily music).** |
| 6 | Gorji, M. A. H., et al. (2014). Effect of auditory stimulation on traumatic coma duration. *Saudi J. Anaesth.*, 8(1), 69-72. | **Intervention = familiar voice (not music).** |
| 7 | Çevik, K., & Namik, E. (2018). Effect of Auditory Stimulation on the Level of Consciousness in Comatose Patients. *J. Neurosci. Nurs.*, 50(6), 375-380. | **Intervention = nurse's voice (not music).** |
| 8 | Hoseinzadeh, E., et al. (2017). Effect of auditory stimulation on consciousness in coma patients with head injury. *J. Nurs. Midwifery Sci.*, 4(4), 82-88. | **Intervention = nurse's voice (not music).** |
| 9 | Liu, Z. B., et al. (2022). Short-term efficacy of music therapy combined with α binaural beat therapy in disorders of consciousness. *Front. Psychol.*, 13, 947861. | **Combined intervention (music + binaural beat), cannot isolate music effect.** |
| 10 | Varghese, R., et al. (2021). Effectiveness of voice stimulus on the level of consciousness, physiological parameters and behavioural responses in comatose patients. *Clin. Epidemiol. Glob. Health*, 9, 150-156. | **Intervention = voice stimulus (not music).** |
| 11 | 袁建, & 刘华. (2022). 正中神经电刺激配合音乐疗法治疗脑外伤后昏迷的临床效果.*实用临床医学*, (6), 9-11. | **Combined intervention (median nerve electrical stimulation + music), cannot isolate music effect.** |
| 12 | 蒋芙蓉, & 冯芳. (2011). 振动音乐疗法结合呼唤对脑出血后昏迷患者的促醒作用.*当代护士(下旬刊)*, (10), 104-106. | **Combined intervention (vibrational music + calling), cannot isolate music effect.** |
| 13 | 黄的, & 徐斌. (2016). 早期高压氧联合音乐疗法对脑外伤昏迷促醒及预后的效果.*包头医学院学报*, 32(6), 2. | **Combined intervention (hyperbaric oxygen + music), cannot isolate music effect.** |
| 14 | 顾彩萍, 等. (2016). 语言呼唤联合音乐促醒方案对重型创伤性脑损伤昏迷病人清醒时间及治疗效果的影响.*护理研究*, 30(28), 3486-3490. | **Combined intervention (voice + music), cannot isolate music effect.** |
| 15 | 葛海娇, 等. (2009). 语言呼唤联合音乐促醒法在重型颅脑损伤昏迷患者中的应用.*现代临床护理*, 8(3), 4. | **Combined intervention (voice + music), cannot isolate music effect.** |
| 16 | 黄的, 等. (2016). 音乐疗法配合高压氧治疗对脑外伤后昏迷促醒的临床观察.*中国康复*, 31(1), 70-71. | **Combined intervention (hyperbaric oxygen + music), cannot isolate music effect.** |
| 17 | 黄丽英. (2018). 音乐疗法联合针灸早期介入对脑出血后昏迷患者促醒效果的临床观察.*中西医结合心血管病电子杂志*, 6(34), 180-181. | **Combined intervention (acupuncture + music), cannot isolate music effect.** |
| 18 | 王飞, 等. (2013). 音乐疗法联合针刺对重度颅脑损伤患者意识提高的疗效观察.*河北中医*, 35(1), 31-32. | **Combined intervention (acupuncture + music), cannot isolate music effect.** |
| 19 | 原文进, 杜晓峰, & 肖非. (2010). 音乐疗法联合盐酸纳洛酮治疗脑出血后昏迷患者疗效观察.*临床医药实践*, 19(11), 461-463. | **Combined intervention (naloxone + music), cannot isolate music effect.** |
| 20 | 陈媛媛, 等. (2024). 音乐疗法联合五感促醒疗法在颅脑损伤术后昏迷患者护理中的应用.*中华养生保健*, 42(18), 150-153. | **Combined intervention (five-sense awakening + music), cannot isolate music effect.** |
| 21 | 臧珍珍, 等. (2022). 音乐疗法联合四感联合促醒刺激护理对高血压性脑出血术后昏迷病人苏醒时间、神经功能及临床预后的影响. *全科护理*, 20(4), 523-525. | **Combined intervention (four-sense stimulation + music), cannot isolate music effect.** |
| 22 | 李华玲, 等. (2022). 音乐疗法联合ACS技术在轻度意识障碍患者磁共振检查中的应用效果.*神经损伤与功能重建*, 17(8), 488-489. | **Combined intervention (ACS technology + music); not a treatment study.** |
| 23 | 李金燕, 等. (2016). 音乐联合抚触唤醒对动脉瘤性蛛网膜下腔出血后脑性昏迷的意识状态及神经功能的影响.*中国临床研究*, 29(9), 1278-1281. | **Combined intervention (touch awakening + music), cannot isolate music effect.** |
| 24 | 鲍秋丽, 等. (2011). 音乐联合抚触对颅脑损伤昏迷患者脑电活动及听觉诱发电位的影响.*护理学杂志*, 26(22), 26-27. | **Combined intervention (touch + music), cannot isolate music effect.** |
| 25 | 洪梦琪, 等. (2024). 选择性音乐疗法联合重复经颅磁刺激对颅脑损伤后意识障碍患者的促醒疗效.*浙江临床医学*, 26(11), 1655-1657. | **Combined intervention (rTMS + music), cannot isolate music effect.** |
| 26 | 任江艳. (2022). 醒脑复苏针刺法联合音乐和光电刺激对持续性植物状态患者的促醒作用研究.*中国民间疗法*, 30(8), 55-58. | **Combined intervention (acupuncture + music + photoelectric stimulation), cannot isolate music effect.** |
| 27 | 杨美琪. (2024). 触觉刺激联合音乐疗法对脑创伤昏迷患者的促醒作用研究. 华北理工大学硕士学位论文. | **Combined intervention (tactile stimulation + music), cannot isolate music effect.** |
| 28 | 王鹤, & 张丹. (2019). 脑外伤后昏迷促醒应用音乐疗法配合高压氧治疗的临床观察研究.*临床医药文献电子杂志*, 6(59), 31. | **Combined intervention (hyperbaric oxygen + music), cannot isolate music effect.** |
| 29 | 饶小英. (2020). 脑出血后昏迷患者采取振动音乐疗法结合呼唤的促醒效果分析.*基层医学论坛*, 24(30), 4355-4356. | **Combined intervention (vibrational music + calling), cannot isolate music effect.** |
| 30 | 金璇, 等. (2022). 四感刺激联合振动音乐疗法在脑出血患者中的应用效果. *中国民康医学*, 34(3), 14-16. | **Combined intervention (four-sense stimulation + vibrational music), cannot isolate music effect.** |
| 31 | 宓洪挺, 等. (2023). 不同情绪特征的音乐联合经颅磁刺激对意识障碍患者神经功能恢复的评估.*中国康复*, 38(8), 483-485. | **Combined intervention (TMS + music), cannot isolate music effect.** |
| 32 | 张君洁, 等. (2025). 亲人音乐干预辅助多维感官促醒刺激对高血压脑出血术后昏迷患者的影响.*医学理论与实践*, 38(18), 3211-3214. | **Combined intervention (multisensory stimulation + music), cannot isolate music effect.** |
| 33 | 闫杰. (2024). HD-tDCS联合音乐刺激对微意识状态患者意识水平的影响研究. 山东中医药大学硕士学位论文. | **Combined intervention (HD-tDCS + music), cannot isolate music effect.** |
| 34 | 林晶. (2013). 系统听觉刺激疗法对颅脑损伤持续植物状态患者的影响.*护理学杂志*, 28(2), 38-39. | **Intervention = auditory stimulation (not specifically music).** |
| 35 | 莫冬梅. (2013). 亲人音乐疗法对脑梗死昏迷患者促醒效果的影响.*当代护士(中旬刊)*, (12), 16-17. | **Combined intervention (family voice + music), cannot isolate music effect.** |
| 36 | 郝习君, 等. (2024). 自然之声音乐疗法对脑创伤昏迷患者的促醒作用.*华北理工大学学报(医学版)*, 26(3), 233-238. | **Intervention = nature sounds (not music).** |

**Ineligible population (n=7)**

| # | Reference | Exclusion reason |
| --- | --- | --- |
| 1 | Segura, E., et al. (2024). Enriched music-supported therapy for individuals with chronic stroke. *J. Neurol.*, 271(10), 6606-6617. | **Population = chronic stroke, not DoC.** |
| 2 | van Bruggen-Rufi, M. C., et al. (2017). The effect of music therapy in patients with Huntington's disease. *J. Huntingtons Dis.*, 6(1), 63-72. | **Population = Huntington's disease, not DoC.** |
| 3 | Moschonas, E. H., et al. (2023). Efficacy of a music-based intervention in a preclinical model of traumatic brain injury. *Exp. Neurol.*, 369, 114544. | **Animal study.** |
| 4 | Golubovic, J., et al. (2025). A randomized pilot and feasibility trial of live and recorded music interventions for management of delirium symptoms in acute geriatric patients. *BMC Geriatr.*, 25(1), 306. | **Population = delirium, not DoC.** |
| 5 | 苏慧霞, 陈艳, & 杨欣. (2020). 早期促醒康复干预对重症病毒性脑炎伴植物状态病儿临床预后的影响. *安徽医药*, 24(1), 20-23. | **Population = pediatric (children), not adult DoC.** |
| 6 | 余育剑, 等. (2022). 音乐辅助疗法对重症颅脑损伤患者的应用效果研究. *基层医学论坛*, 26(21), 7-9. | **No clear diagnosis of DoC (mixed population).** |
| 7 | 黄莉, 等. (2007). 重型颅脑损伤昏迷患者音乐疗法24例效果观察. *齐鲁护理杂志*, (12), 23. | **No extractable quantitative consciousness outcome (composite score with unclear scale).** |

**Ineligible measure & outcome (n=16)**

*Note: Studies that did not report a valid, extractable quantitative outcome on consciousness level using standardized DoC assessment tools (e.g., CRS‑R, GCS), or lacked a control group, or used non‑behavioral/electrophysiological outcomes, or had incomplete data reporting.*

| # | Reference | Exclusion reason |
| --- | --- | --- |
| 1 | Steinhoff, N., et al. (2015). A pilot study into the effects of music therapy on different areas of the brain of individuals with unresponsive wakefulness syndrome. *Front. Neurosci.*, 9, 291. | **Outcome = PET tracer uptake (cerebral metabolism); no behavioral consciousness scale (e.g., CRS‑R or GCS) reported.** |
| 2 | Puggina, A. C. G., da Silva, M. J. P., & Santos, J. L. F. (2011). Use of music and voice stimulus on patients with disorders of consciousness. *J. Neurosci. Nurs.*, 43(1), E8‑E16. | **Earlier publication of the same study as Puggina & da Silva (2015) (which was included); primary outcomes were vital signs and facial expressions, not consciousness level; excluded to avoid duplicate data.** |
| 3 | 李节惠. (2023). 基于近红外光谱成像技术探索音乐刺激对慢性意识障碍患者大脑皮层血流动力学的影响. 山东中医药大学硕士学位论文. | **Outcome = cerebral hemodynamics measured by fNIRS (oxygenated hemoglobin signal); no behavioral consciousness scale (CRS‑R/GCS) used as outcome measure.** |
| 4 | 侯立群, 等. (2015). 严重意识障碍患者对音乐和嗜好刺激的EEG研究. *杭州电子科技大学学报(自然科学版)*, 35(4), 93‑96. | **Outcome = EEG wavelet energy values; no behavioral consciousness scale reported.** |
| 5 | 韩雪玲. (1991). 脑外伤后意识障碍的音乐效应. *中华护理杂志*, (3), 111‑112. | **Case series (n=8) without a control group; no standardized consciousness scale used; only descriptive outcomes.** |
| 6 | 徐树平, 等. (2019). 音乐治疗在老年脑梗死意识障碍患者中的应用. *广东医学*, 40(2), 308‑310. | **Outcome measured using the consciousness subscale of the NIHSS, which is not a validated DoC‑specific assessment tool (standard tools are CRS‑R or GCS).** |
| 7 | 朱春梅, 等. (2019). 音乐疗法在脑出血意识障碍患者中的应用研究. *护士进修杂志*, 34(2), 166‑168. | **Outcome measured using the consciousness subscale of the NIHSS (same issue as #6).** |
| 8 | 陈春瑞, & 王红娟. (2011). 音乐疗法在神经外科昏迷患者催醒实施中的疗效观察. *当代护士(下旬刊)*, (2), 54‑55. | **No quantitative consciousness scores reported (only the number of patients who regained consciousness and the number who improved, without baseline‑to‑endpoint GCS or CRS‑R data).** |
| 9 | 梁颖. (2005). 音乐疗法在神经外科昏迷病人中的应用. *蛇志*, (4), 278‑279. | **No control group; no extractable quantitative outcome data.** |
| 10 | 梁飞凤. (2008). 音乐疗法在神经外科昏迷病人中的疗效观察. *中国医药导报*, (13), 146‑147. | **Same study as #8 (identical abstract); no extractable GCS or CRS‑R data.** |
| 11 | 王金英, & 刘玮. (2003). 音乐疗法用于颅脑手术后昏迷效果观察. *山东医药*, (20), 69‑70. | **No control group; only descriptive outcomes (no GCS scores reported).** |
| 12 | 贾杰, 陈希源, & 于晓忠. (2013). 音乐疗法对脑出血患者意识障碍的疗效影响. *陕西中医*, 34(6), 662‑663. | **Incomplete reporting: states that GCS scores differed significantly between groups at 4 weeks, but no means, SDs, or exact statistics are provided.** |
| 13 | 李爱琴, 等. (2019). 音乐疗法对ICU颅脑损伤意识障碍（Doc）患者意识的影响. *中国现代医生*, 57(34), 87‑89. | **Incomplete reporting: states that GCS and CRS‑R scores were significantly higher in the intervention group, but no means, SDs, or extractable effect sizes are provided.** |
| 14 | 乐美芬, 等. (2001). 音乐对颅脑损伤意识障碍早期促醒效应的探讨. *上海护理*, (4), 1‑3. | **Outcome = BIS (bispectral index) and SEF (spectral edge frequency), which are electrophysiological parameters, not a standardized behavioral consciousness scale.** |
| 15 | 戴敏超, 等. (2016). 选择性音乐疗法对脑外伤意识障碍患者的康复促醒疗效研究. *中国现代医学杂志*, 26(22), 64‑67. | **Incomplete reporting: states that music therapy improved GCS scores, but no means, SDs, or extractable effect sizes are provided.** |
| 16 | 黄莉, 等. (2007). 重型颅脑损伤昏迷患者音乐疗法24例效果观察. *齐鲁护理杂志*, (12), 23. | **Composite outcome comprising 10 indicators (including vital signs, facial expressions, eye opening, and GCS); GCS not reported separately, and no extractable consciousness‑specific data available.** |

**Unable to get full text (n=4)**

| # | Reference | Exclusion reason |
| --- | --- | --- |
| 1 | Sobeeh, M., Öztürk, G., & Hamed, M. (2019). Effect of listening to high arousal music with different valences on reaction time and interference control: Evidence from Simon task. *IBRO Reports*, 6, S441. | **Conference abstract only; full text not available after multiple attempts.** |
| 2 | Rahimi, F., Salehi, K., & Seidi, J. (2019). The effect of pleasant audio stimulation on the level of consciousness of comatose patient: A randomized clinical trial. *Acta Med. Mediterr.*, 35(2), 985-997. | **Full text not accessible.** |
| 3 | Yaghoubinia, F., et al. (2016). Effect of music therapy and reflexology on pain in unconscious patients. *Int. J. Med. Res. Health Sci.*, 5(9), 288-295. | **Full text not accessible.** |
| 4 | Lyu, J., et al. (2014). The effect of music therapy on memory, language and psychological symptoms of patients with mild Alzheimer's disease. *Chin. J. Neurol.*, 47(12), 831-833. | **Population = Alzheimer's disease, not DoC; also full text not available.** |

**Summary of excluded studies:**

Ineligible setting: 5

Ineligible intervention: 36

Ineligible population: 7

Ineligible measure & outcome: 16

Unable to get full text: 4

Total unique excluded studies: 68

Note on related studies:

Puggina (2011) and Puggina & da Silva (2015) report on the same patient cohort; the 2015 version was included because it provided complete data for meta-analysis, while the 2011 version is excluded to prevent duplicate data.

Şirin Gök et al. (2025) was excluded due to its within-subject (cross-over) design, which is incompatible with the parallel-group meta-analysis framework used in this study.
